# Beyond CD30: Dual‐Targeting of Malignant and Regulatory T Cells by Brentuximab Vedotin Remodels the Lymphoma Microenvironment and Overcomes Resistance via BCL2 Inhibition in Mycosis Fungoides

**DOI:** 10.1002/advs.202517353

**Published:** 2026-02-28

**Authors:** Yi Jiang, Pan Lai, Mingjia Li, Yujie Wen, Mengzhou Cao, Yu Xiao, Zhuojing Chen, Jingru Sun, Yang Wang

**Affiliations:** ^1^ Department of Dermatology and Venereology Peking University First Hospital Beijing China; ^2^ National Clinical Research Center for Skin and Immune Diseases Beijing China; ^3^ NMPA Key Laboratory for Quality Control and Evaluation of Cosmetics Beijing China; ^4^ Peking‐Tsinghua Center for Life Sciences Peking University Beijing China

**Keywords:** BCL2, brentuximab vedotin, interferon response, mycosis fungoides, tumor microenvironment

## Abstract

Mycosis fungoides (MF), the most common subtype of cutaneous T cell lymphoma (CTCL), has a poor prognosis in advanced stages. Brentuximab vedotin (BV), a CD30‐targeting antigen‐drug conjugate approved for CD30^+^ MF following prior systemic treatment, still exhibits resistance with unclarified mechanisms. With single‐cell RNA analysis on 13 paired tumor samples from 6 CD30^+^ MF patients, we revealed that BV‐induced immunogenic cell death (ICD) in both CD30^+^ and CD30^−^ malignant T cells while specifically enhanced interferon‐α (IFNα) and IFNγ responses in CD30^−^ subsets. BV also directly targeted CD30^+^ tumor‐infiltrating regulatory T cells (TI‐Tregs), and activated anti‐tumor immunity mediated by dendritic cells and CD8^+^ T cells. The treatment responses and mechanistic insights were validated using seven CTCL cell lines. Resistance arose from upregulated drug efflux transporters and impaired endosomal processing in CD30^+^ malignant T cells, while CD30^−^ tumor cells showed blunted IFNα and IFNγ responses. Anti‐apoptotic BCL2 was upregulated in all tumor cells from nonresponsive lesions, especially in CD30^−^ subsets. We further confirmed a potent synergy between BV and BCL2 inhibitors in tumor cell lines, indicating a promising strategy to overcome resistance in CTCL.

## Graphical abstract

Single‐cell RNA analyses of paired lesions from CD30^+^ mycosis fungoides patients demonstrate that brentuximab vedotin (BV) induces immunogenic cell death in both CD30^+^ and CD30^−^ malignant T cells. BV also targets regulatory T cells and remodels tumor microenvironment, while resistance is driven by impaired IFN responses, drug efflux, and BCL2 upregulation. Combined BV and BCL2 inhibition show synergistic anti‐tumor activity.

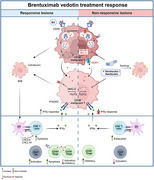

## 1 Introduction

Cutaneous T cell lymphoma (CTCL) represents a heterogeneous group of malignancies derived from mature T cells that primarily reside in the skin. Mycosis fungoides (MF), the most common subtype of CTCL, manifests as skin patches, plaques, and tumors, and progresses slowly over years in most cases [1, 2]. However, 9–34% of patients progress to advanced stages, with poor prognosis, and relapsed or refractory (R/R) to conventional therapies [3, 4, 5]. Moreover, 20–55% of MF patients develop large cell transformation (MF‐LCT), characterized by rapidly progressive skin tumors, aggressive extracutaneous spreading with a 5 year survival of 38.5% [6, 7]. Currently, the lack of effective treatments for MF‐LCT patients prompts an urgent need to pursue more effective therapies.

Brentuximab vedotin (BV) is an antibody‐drug conjugate (ADC) that targets CD30, a member of the tumor necrosis factor receptor superfamily overexpressed on tumor cells across 48–100% MF patients, especially in patients with LCT [8, 9]. BV combines the monoclonal antibody (brentuximab) with the antitubulin agent monomethyl auristatin E (MMAE) through a protease‐cleavable linker [10]. After binding to CD30 on tumor cells, BV is internalized, and cleaved by the lysosome, leading to the intracellular release of MMAE, which induces cell cycle arrest and therefore apoptosis [11, 12, 13]. Clinically, BV has shown promising efficacy and tolerability in MF patients and is approved as monotherapy for adult patients with CD30^+^ MF who have received prior systemic therapy [14, 15, 16]. However, favorable responses were also found in patients with low or negative expression levels of CD30, while the action mechanisms of BV in CD30^+^ MF have not been fully clarified [17, 18]. Notably, coexistence of both responsive and nonresponsive lesions in the same patient has also been observed in our clinical practice, independent of their overall outcomes. Moreover, despite encouraging response rate, most patients suffer progressive disease or refractory and only a minority of complete responses to BV appear durable, while the underlying mechanisms remain undetermined [19, 20, 21].

**FIGURE 1:**
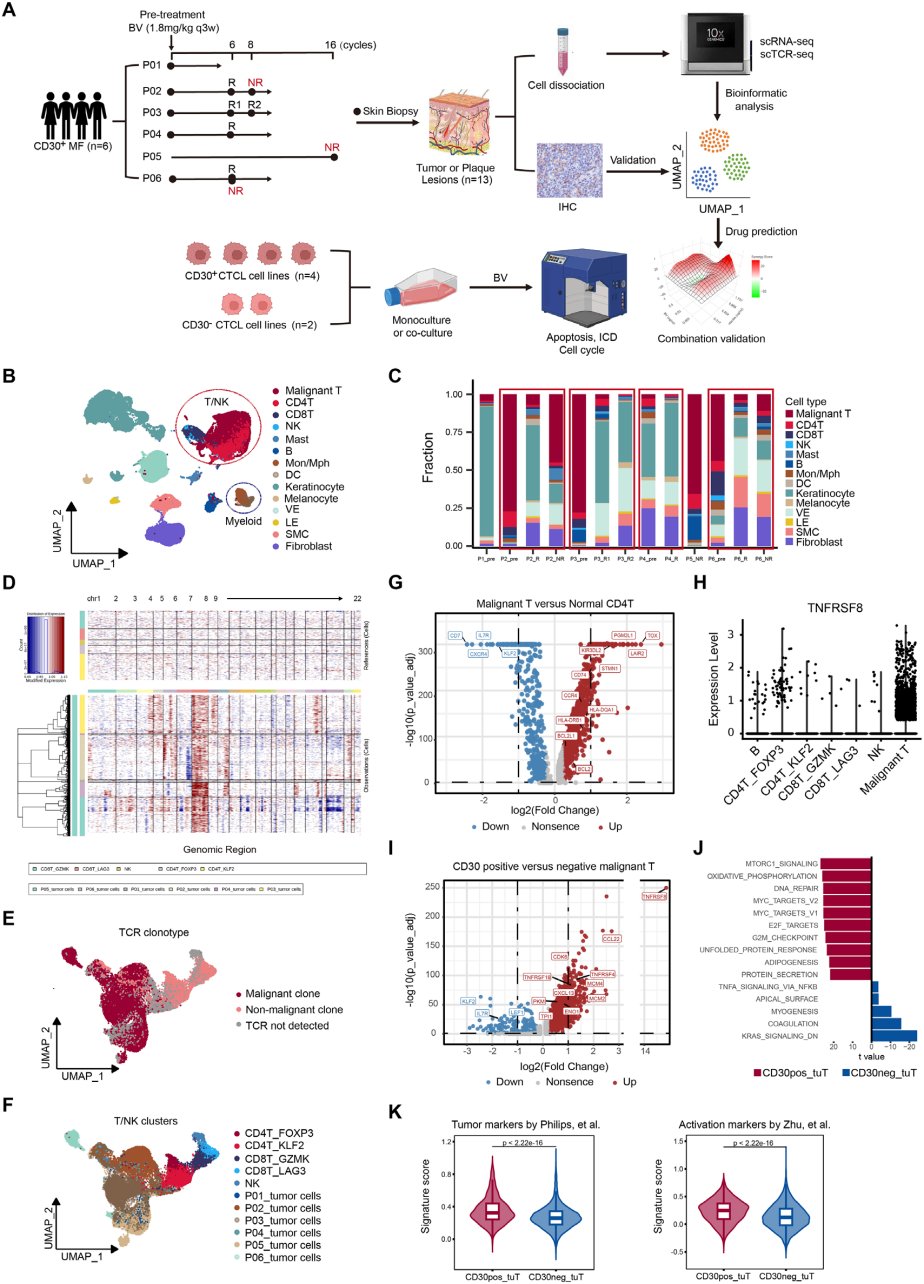
Single‐cell transcriptional profiling of 13 MF samples during BV treatment. (A) Scheme of the overall study design. (B) Uniform manifold approximation and projection (UMAP) plot of all cells colored by major cell types according to canonical markers. (C) Bar plots indicating the proportion of major cell types in each patient. Paired samples were marked by red box. (D) Large‐scale copy number variations (CNVs) of single cells from all samples. (E,F) UMAP plot of all T/natural killer (NK) cells after reclustering, with cells colored by TCR information (E) and clusters (F). Malignant T cells are clustered by patient ID with the suffix “_tumor cells”. (G) Volcano plot showing the differentially expressed genes between malignant T cells and normal CD4^+^ T cells (CD4T_FOXP3 were excluded). (H) Expression of CD30 in lymphoid cells. (I) Volcano plot showing the differentially expressed genes between CD30^+^ and CD30^−^ malignant T cells pretreatment. (J) Top 10 differences in pathway activities scored per cell by gene set variation analysis (GSVA) comparing CD30^+^ malignant T cells (*n* = 779) and CD30^−^ malignant T (*n* = 12,569) pretreatment. (K) Violin plots showing signature scores of tumor markers (left), and activation (right) for CD30^+^ and CD30^−^ malignant T cells pretreatment. Two‐tailed unpaired Wilcoxon rank‐sum test was used.

In this study, we illustrated the dynamics of CD30^+^ and CD30^−^ tumor cells and the tumor microenvironment (TME) during treatment by single‐cell RNA sequencing (scRNA‐seq) and T cell receptor sequencing (scTCR‐seq) on paired lesions from CD30^+^ MF patients treated with BV. We demonstrated the common and differential responses of CD30^+^ and CD30^−^ tumor cells to BV, as well as the features of nonresponsive lesions and investigated combination strategies to counteract their resistance toward BV. The research flow of our study is shown in Figure 1A.

## 2 Results

### 2.1 Single‐Cell Atlas Demonstrated That CD30^+^ Malignant T Cells in MF Exhibited Highly Proliferative and Aggressive Features

We prospectively collected 13 tumor samples from 6 patients with clinically confirmed CD30^+^ MF for analysis by scRNA‐seq and scTCR‐seq (Figure 1A). All patients had histologic diagnosis of advanced‐stage (IIB to IVA2) MF‐LCT. CD30 was detected in all pretreatment samples, with the proportion of positive cells within the samples ranging from 12% to 26% by immunohistochemistry (IHC) (Table S1). Eight posttreatment samples were collected from skin biopsies following 6, 8, or 16 cycles (3 weeks per cycle) of BV treatment according to the reported time to response [16, 17]. Five patients achieved partial response, and one had stable disease toward BV treatment. Despite overall therapeutic response, three of these six patients concurrently developed new lesions or had unresolved lesions during treatment. Based on clinical and pathologic assessment, the posttreatment samples were categorized into two groups: major pathologic responsive (R, *n* = 5) and nonresponsive lesions (NR, *n* = 3). The three nonresponsive lesions included one newly developed lesion from P02 (P2_NR) after 8 cycles of treatment and two persistent lesions from P05 (P5_NR) and P06 (P6_NR) following 16 and 6 cycles of treatment, respectively. Patient characteristics are listed in Table S1.

After quality control and the removal of doublets, a total of 97 044 cells with a median of 1732 genes per cell were retained. Unsupervised clustering identified 24 clusters (Figure S1A), and no significant batch effects were observed across patients or response groups (Figure S1B,C). The average number of genes and unique molecular identifiers were comparable among the different clusters (Figure S1D). Based on the expression of well‐established gene markers, the 24 clusters were annotated as B cells, T cells (including CD4^+^ T, CD8^+^ T, and malignant T cells), natural killer (NK) cells, myeloid cells (including dendritic cells (DCs), monocytes, and macrophages), mast cells, and skin‐resident nonimmune cells (Figure 1B,C; Figure S1E).

Malignant T cells were monoclonal, characterized by abnormal copy number variations (CNVs), especially amplifications on chromosome 7 (Figure 1D,E). The intertumor heterogeneity of malignant T cells was evident in the uniform manifold approximation and projection (UMAP) plot of T/NK cells, whereas normal T cells defined by their canonical markers, including CD4^+^ memory T cells (Tm, CD4T_KLF2), CD4^+^ tumor‐infiltrating regulatory T cells (TI‐Tregs, CD4T_FOXP3) and CD8^+^ T cells, showed substantial overlap across patients (Figure 1F; Figure S1F). Consistent with previous studies, malignant T cells demonstrated CD7 loss and upregulation of genes, such as *TOX* and *KIR3DL2* compared to normal CD4^+^ T cells (Figure 1G; Table S2).

CD30 was most highly expressed by malignant T cells, followed by TI‐Tregs (Figure 1H). To characterize the molecular features of CD30^+^ malignant T cells, we analyzed the gene expression profiles of all malignant T cells before BV treatment. Compared to their CD30^−^ counterparts, CD30^+^ malignant T cells highly expressed genes involved in cell proliferation, (*CDK6*, *MCM2*, *MCM4*), glycolysis (*PKM*, *ENO1*, *TPI1*), and oxidative phosphorylation (*NDUFS6*, *NDUFB2*, *ATP5MC3*) (Figure 1I,J; Table S3). Gene set variation analysis (GSVA) further revealed that oncogenic pathways were enriched in CD30^+^ malignant T cells, including the mTORC1, Myc, and E2F signaling pathways (Figure 1J). CD30^+^ malignant T cells also showed an increased MF tumor score defined by Philips, et al. [22] and exhibited enhanced activation compared to CD30^−^ malignant T cells (Figure 1K). Collectively, these findings indicate that CD30^+^ malignant T cells possess more active and aggressive characteristics.

### 2.2 BV Induces Immunogenic Cell Death in all Malignant T Cells and Enhances Interferon Signaling in CD30^−^ Subsets

**FIGURE 2:**
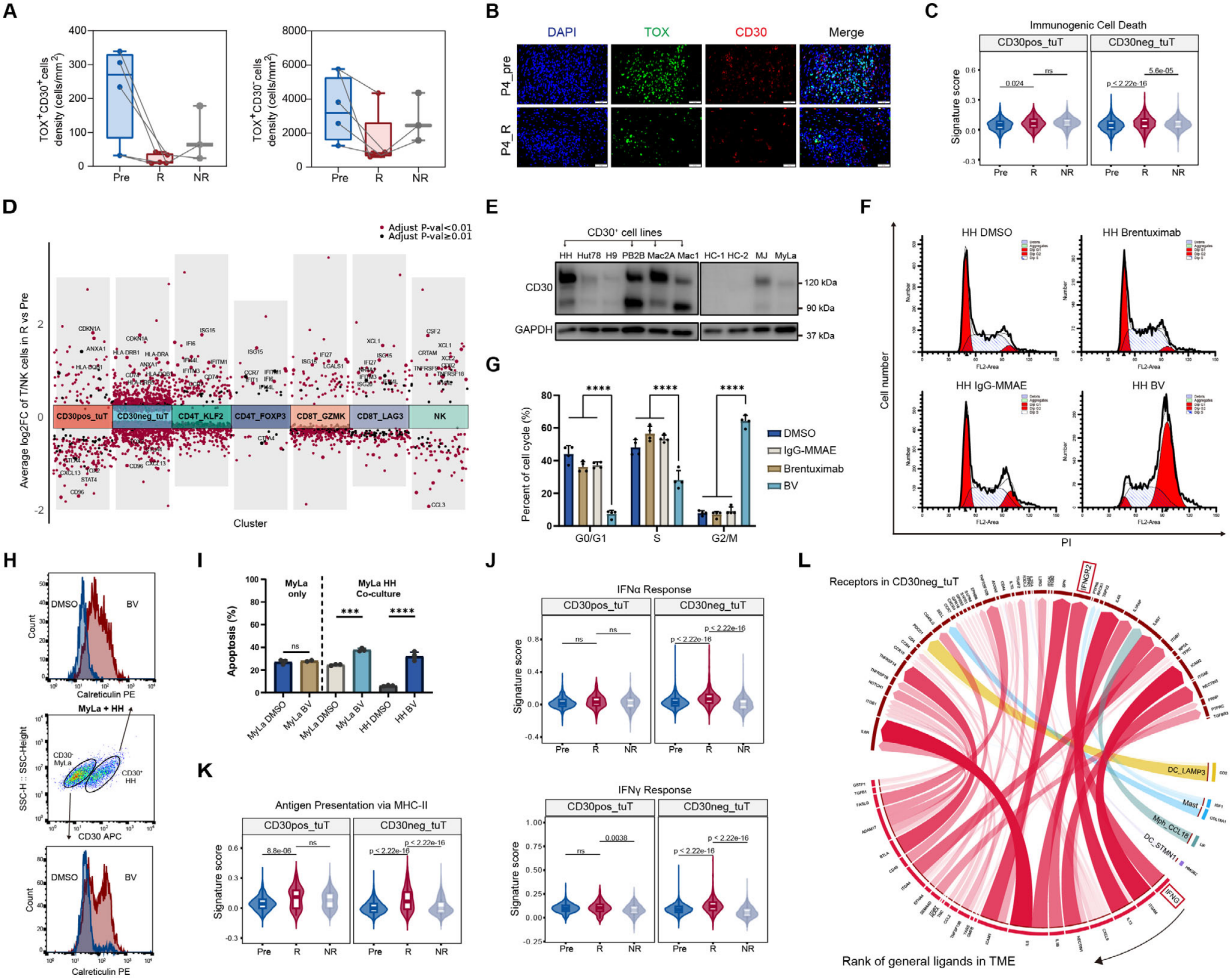
Transcriptomic and functional remodeling of malignant T cells following BV treatment. (A) Boxplot showing the cell density of TOX^+^CD30^+^ (left) and TOX^+^CD30^−^ (right) malignant T cells of each sample based on multiplex IHC staining in Pre (*n* = 4), R (*n* = 5), NR (*n* = 3) groups. Paired samples were linked with gray lines. Two‐tailed paired Wilcoxon signed‐rank test was used. (B) Representative multiplex IHC staining of TOX (green) and CD30 (red) on paired samples. 4′‐6′‐diamidino‐2‐phenylindole (DAPI) (blue) was used to visualize cell nuclei. Scale bar = 50 µm. (C) Violin plots showing signature scores of immunogenic cell death for CD30^+^ and CD30^−^ malignant T cells in Pre, R, and NR groups, respectively. Two‐tailed unpaired Wilcoxon rank‐sum test was used. (D) Differentially expressed genes between R and Pre groups across all T/NK clusters. (E) CD30 protein expression of CTCL cell lines and normal CD4^+^ T cells from peripheral blood of two healthy controls (HC). (F,G) Changes of the G0/G1, S, and G2/M population of HH treated by BV and control agents via PE cell‐cycle analysis. One‐way ANOVA followed by Dunnett's post hoc test was used. (H) Flow cytometry analysis of cell surface expression of calreticulin (CRT) in CD30^+^ HH and CD30^−^ MyLa under coculture condition. Unpaired *t*‐test was used. (I) Apoptosis rates of MyLa treated by BV or DMSO in mono‐ and coculture with HH. Unpaired *t*‐test was used. (J,K) Violin plots showing signature scores of interferon‐α (IFNα) and IFNγ responses (J) and antigen presentation via MHC‐II (K) for CD30^+^ and CD30^−^ malignant T cells in Pre, R, and NR groups, respectively. Two‐tailed unpaired Wilcoxon rank‐sum test was used. (L) Circos plot showing links between predicted ligands from sender cells (bottom) with their associated receptors on receiver cells (top). Width and transparency of the blocks represent the prior interaction weight of the ligand–receptor interactions. Colors on the lower part of the plots represent cell types where ligands are mostly derived from: dark red, ligands expressed in multiple cell types (general); orange, DC_LAMP3; blue, mast cells; green, Mph_CCL18; purple, DC_STMN1. ***p < 0.001, ****p < 0.0001.

BV treatment significantly decreased the tumor burden in responsive lesions, as evidenced by decreased modified Severity‐Weighted Assessment Tool (mSWAT) scores and marked improvement of skin lesions at week 18 (after 6 cycles) in all patients (Figure 1C; Table S1, and Figure S2). Consistent with previous studies, treatment led to reduced CD30 expression in skin lesions, as revealed by scRNA‐seq and further confirmed by IHC (Figure S3A) [23, 24]. This concordance also supports the reliability of our scRNA‐seq‐based analysis for treatment‐associated changes. However, the proportion of CD30^+^ malignant T cells within the tumor cell population by single cell analyses did not show consistent decreases across paired samples (Figure S3B). Given the confirmed overexpression of *TOX* in malignant T cells from patients, *TOX* was used as a marker for malignant T cells in multiplex IHC analyses. This analysis demonstrated that the densities of both CD30^+^ and CD30^−^ malignant T cells were decreased in responsive lesions (Figure 2A,B). Notably, this decrease was consistently observed across all matched pretreatment and responsive samples, although statistical significance was not reached due to the limited number of paired cases. These results indicate that both CD30^+^ and CD30^−^ malignant T cells were reduced following BV treatment in responsive lesions. Consistently, further single cell analyses demonstrated that upon treatment, both CD30^+^ and CD30^−^ malignant cells underwent apoptosis with cell cycle arrest and increased G2M scores, and immunogenic cell death (ICD) with enhanced endoplasmic reticulum stress (Figure 2C; Figure S3C). ICD is a functional response pattern of stress‐induced regulated cell death that can elicit adaptive immune responses through the release of damage‐associated molecular patterns (DAMPs) from dying cells, such as calreticulin (CRT), annexin A1 (ANXA1), heat‐shock proteins (HSPs), and high mobility group protein B1 (HMGB1) [25]. Consistently, both CD30^+^ and CD30^−^ malignant T cells showed significantly increased expression of *CDKN1A* (encoding p21, a cyclin‐dependent kinase (CDK) inhibitor inducing cell cycle arrest and apoptosis [26, 27]) and ICD markers after treatment, including *ANXA1*, *HMGB1*, and several HSPs (Figure 2D; Table S4).

To determine whether CD30^−^ malignant T cells were BV's direct targets or if they were affected by bystander effect, we cocultured CD30^+^ and CD30^−^ CTCL cells to confirm the effect of BV treatment (Figure 2E; Figure S3D). Tumor cell lines were treated with 0.5 or 1 µg/mL of BV, as well as its controls, including dimethyl sulfoxide (DMSO), brentuximab, and IgG‐MMAE at equivalent concentrations for 24–48 h. IgG‐MMAE is a nonbinding, isotype‐control ADC, which shares the same structure as that of BV but replaces the CD30 monoclonal antibody brentuximab with its isotype IgG. Under BV treatment, the CD30^+^ cell line (HH) underwent cell cycle arrest at the G2/M phase and increased surface CRT expression (Figure 2F–H). These effects were not observed with treatment using either brentuximab alone or IgG‐MMAE, confirming that CD30 binding and internalization are required for BV's activity. Intriguingly, in the presence of CD30^+^ malignant T cells, BV treatment‐induced apoptosis and ICD in CD30^−^ cell lines MyLa and Hut78, while they did not respond to BV treatment under monoculture (Figure 2H,I; Figure S3E,F). This finding supports a potent bystander effect, where the MMAE released from BV‐targeted CD30^+^ tumor cells subsequently kills neighboring CD30^−^ tumor cells.

Interestingly, beyond the effects shared with CD30^+^ malignant T cells, CD30^−^ malignant T cells in responsive lesions exhibit a specific upregulation of interferon‐α (IFNα) and IFNγ signaling pathways following BV treatment. Genes involved in the IFNα and IFNγ responses were among the most significantly enriched pathways (Figure 2J; Figure S3G). Correspondingly, several *MHC‐II* genes, well‐established downstream targets of IFNγ signaling, were upregulated in CD30^−^ malignant T cells from responsive lesions (Figure 2K). Studies have shown that MHC‐II expression correlates with immunotherapy response and better prognosis across multiple tumor types [28, 29]. Furthermore, NicheNet analysis [30] of upregulated genes in posttreatment CD30^−^ malignant T cells identified the *IFNG*‐*IFNGR2* interaction as the top active ligand–receptor pair in responsive lesions (Figure 2L; Figure S3H). The source of this IFNγ was primarily the CD8T_LAG3 cluster, a T cell population coexpressing high levels of cytotoxic and exhausted markers that expanded after BV (Figures S1F and S3I). Taken together, while BV‐induced apoptosis and ICD in both CD30^+^ and CD30^−^ tumor cells through direct targeting and bystander effect, respectively, the CD30^−^ subset specifically showed enhanced responsiveness to IFNα and IFNγ within the remodeled tumor microenvironment.

### 2.3 Tumor‐Infiltrating Tregs Are Additional Targets of BV with Attenuated Immunosuppressive Function Posttreatment

**FIGURE 3:**
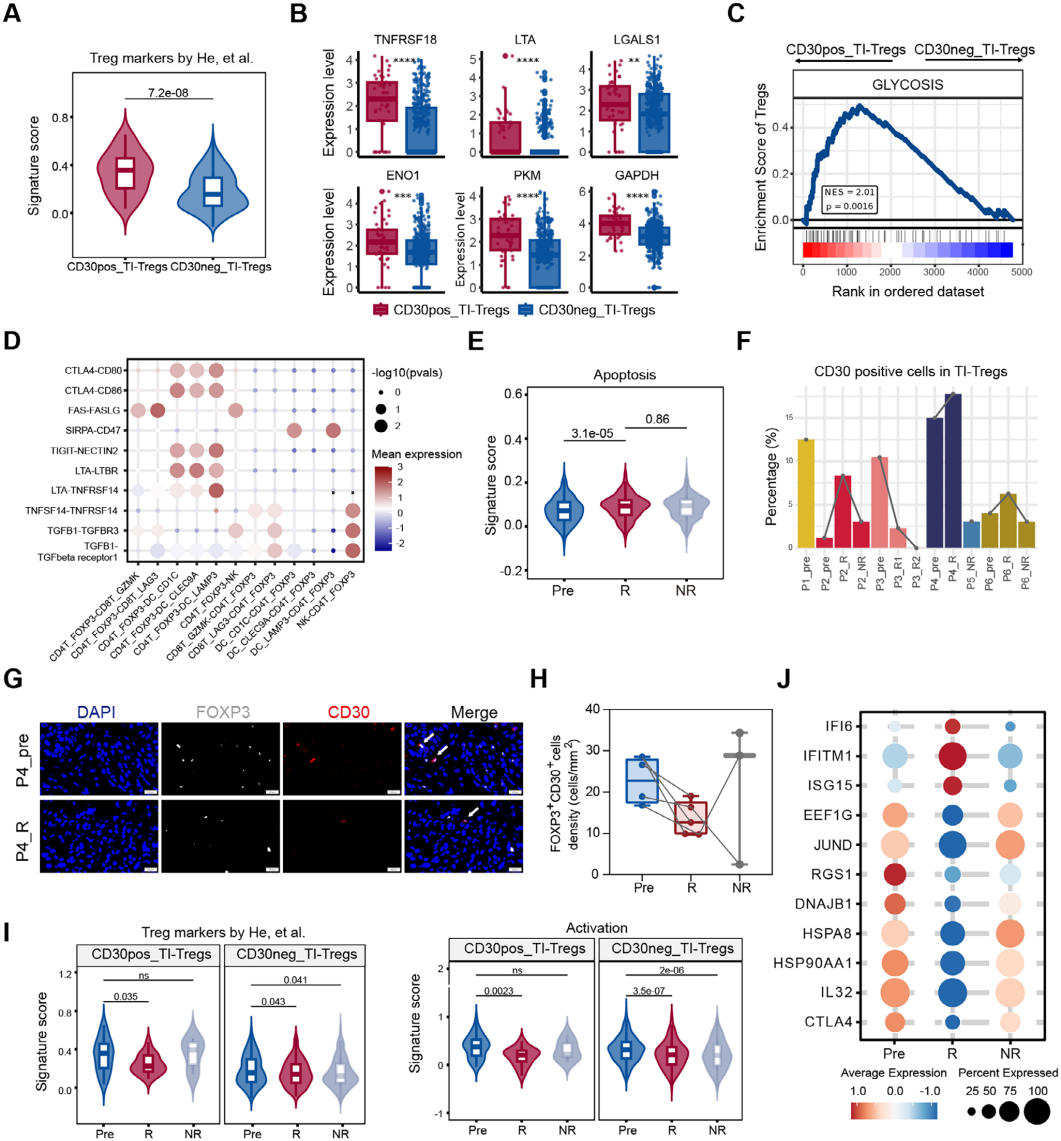
TI‐Tregs as additional target of BV with impaired immunosuppressive function after treatment. (A) Violin plot showing signature scores of Treg phenotype markers of CD30^+^ TI‐Tregs compared to CD30^−^ TI‐Tregs. Two‐tailed unpaired Wilcoxon rank‐sum test was used. (B) Boxplots showing the differentially expressed genes between CD30^+^ and CD30^−^ TI‐Tregs. **p < 0.01, ***p < 0.001, ****p < 0.0001. (C) GSEA analysis found the GLYCOLYSIS was enriched in CD30^+^ TI‐Tregs compared to CD30^−^ TI‐Tregs. (D) Summary of selected ligand–receptor interactions from CellPhoneDB between TI‐Tregs and CD8^+^ T cells, NK cells and DCs in all samples. (E) Violin plot showing signature scores of apoptosis of TI‐Tregs in Pre, R, and NR groups. Two‐tailed unpaired Wilcoxon rank‐sum test was used. (F) Bar plot showing the fraction of CD30^+^ TI‐Tregs of TI‐Tregs from each sample based on mRNA expression levels. Paired samples were linked by gray lines. (G) Multiplex IHC staining of FOXP3 (white) and CD30 (red) on paired samples. DAPI (blue) was used to visualize cell nuclei. CD30^+^ TI‐Tregs are indicated by white arrows. Scale bar = 20 µm. (H) Boxplot showing the cell density of FOXP3^+^CD30^+^ TI‐Tregs of each sample based on multiplex IHC staining in Pre (*n* = 4), R (*n* = 5), NR (*n* = 3) groups. Paired samples were linked with gray lines. Two‐tailed paired Wilcoxon signed‐rank test was used. (I) Violin plots showing signature scores of Treg markers and activation of TI‐Tregs in Pre, R, and NR groups. Two‐tailed unpaired Wilcoxon rank‐sum test was used. (J) Dot plot showing the expression of differentially expressed genes of TI‐Tregs in Pre, R, and NR groups.

Our single‐cell data further revealed that TI‐Tregs were the second most significant CD30‐expressing population (Figure 1H). Previous study demonstrated that the CD25^high^ Treg subset with high CD30 expression possessed stronger suppressive potency and a greater dependence on IL‐2 for TCR‐induced proliferation than the CD25^+^ subset [31]. Accordingly, pretreatment CD30^+^ TI‐Tregs in our cohort exhibited a more pronounced regulatory phenotype than their CD30^−^ counterparts, characterized by the upregulation of genes involved in immunosuppression and glucose metabolism (Figure 3A–C). Analysis of intercellular interactions using by CellPhoneDB [32] revealed extensive suppressive interactions between TI‐Tregs and antitumoral immune cells within the TME (Figure 3D). These observations prompted us to investigate the functional impact and dynamics of TI‐Tregs following BV treatment.

Following BV treatment, the proportion of TI‐Tregs among normal T/NK cells decreased in the majority of patients (Figure S4A). Notably, the apoptosis of TI‐Tregs increased (Figure 3E). Parallel to the pattern observed in malignant T cells, the proportion of CD30^+^ TI‐Tregs within TI‐Tregs by scRNA‐seq did not exhibit a consistent decrease across paired samples after treatment (Figure 3F). Consistently, multiplex IHC analysis demonstrated an absolute decrease in TI‐Tregs in responsive lesions, particularly CD30^+^ TI‐Tregs in all paired samples (Figure S4B; Figure 3G,H), although statistical significance was not reached due to the limited number of paired cases. In responsive lesions, both CD30^+^ and CD30^−^ TI‐Tregs showed attenuated activation and immunosuppressive capacity, as evidenced by the downregulation of *CTLA4* (Figure 3I,J), while the immunosuppressive capacity of CD30^+^ TI‐Tregs in nonresponsive lesions remained comparable to that in pretreatment lesions (Figure 3I). Collectively, these data indicate that tumor‐infiltrating TI‐Tregs are direct targets of BV. Their depletion and functional attenuation represent a key mechanism by which BV remodels the immunosuppressive tumor microenvironment.

### 2.4 BV Reprograms the Tumor Immune Microenvironment to Support Anti‐Tumor Immunity

**FIGURE 4:**
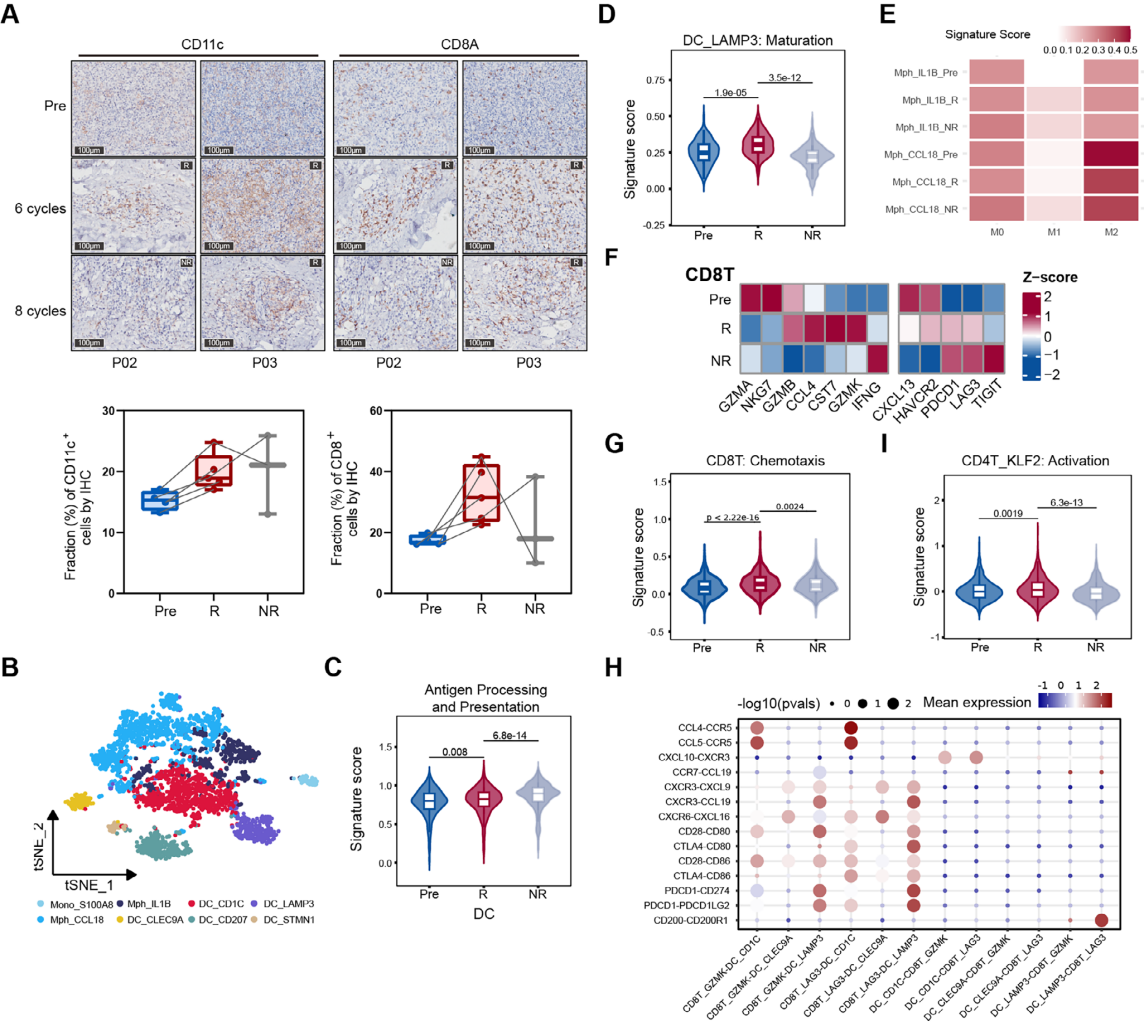
Reprogrammed TME supports the anti‐tumor immunity after BV treatment. (A) Representative images of IHC staining (200×) of canonical surface markers for DCs (CD11c), CD8^+^ T (CD8A) cells in paired lesions from Pre, R, and NR groups of two patients (P02, P03), respectively (top). Quantification of fractions of CD8^+^ T and DCs from the IHC images (bottom). Two‐tailed paired Wilcoxon signed‐rank test was used. (B) UMAP plot of myeloid cells colored by cell clusters according to canonical markers. (C,D) Violin plot showing signature scores of antigen presentation of DCs (C), the maturation of mregDCs (D) in Pre, R, and NR groups. Two‐tailed unpaired Wilcoxon rank‐sum test was used. (E) Heatmap of module scores of M0, M1, and M2 signatures among macrophage clusters. (F) Heatmap of normalized expression of cytotoxic and exhausted genes among CD8^+^ T cells in Pre, R, and NR groups. (G) Violin plot showing signature scores of chemotaxis of CD8^+^ T in Pre, R, and NR groups. Two‐tailed unpaired Wilcoxon rank‐sum test was used. (H) Summary of selected ligand–receptor interactions from CellPhoneDB between CD8^+^ T cells and DCs in R lesions. (I) Violin plot showing signature scores of activation for CD4^+^ memory T cells (CD4T_KLF2) in Pre, R, and NR groups. Two‐tailed unpaired Wilcoxon rank‐sum test was used.

As noted above, BV‐induced ICD in malignant T cells which may activate immune responses and repressed TI‐Tregs. Therefore, we further investigated the alterations in the composition and functional characteristics of the TME following BV treatment. Corresponding to the decrease of malignant T cells in responsive lesions, the proportion of major cell types, including dendritic cells, CD4^+^ Tm cells, CD8^+^ T cells, NK cells increased in most lesions, although the *p* values were not significant due to limited sample size (Figure S5A). IHC staining in skin lesions corresponding to scRNA‐seq samples confirmed the consistently increased infiltration of DCs (CD11c^+^) and CD8^+^ T cells (CD8A^+^) in all responsive lesions (Figure 4A; Figure S5B).

During ICD, the emission of DAMPs from tumor cells and their binding to specific pattern recognition receptors expressed by DCs triggers the maturation of DCs and the subsequent activation of anti‐tumor responses [25]. We identified five subclusters of DCs, including conventional type I DCs (cDC1, DC_CLEC9A), cDC2 (DC_CD1C), Langerhans cells (LC, DC_CD207), mature DCs enriched in immunoregulatory molecules (mregDC, DC_LAMP3), and a small subset of proliferating DCs (DC_STMN1) (Figure 4B; Figure S5C). After BV treatment, the overall antigen‐presenting signature of DCs was significantly enhanced in both responsive and nonresponsive lesions (Figure 4C). mregDCs had the lowest stemness score and could be generated from other DCs in trajectory analysis (Figure S5D,E) [33, 34]. In our patients, the maturation status of mregDCs was further induced in responsive lesions, accompanied by a reduction in their coinhibition effects on effector cells, thereby conferring anti‐tumor activity (Figure 4D; Figure S5F). Moreover, two macrophage subtypes were identified, including immunosuppressive M2‐like Mph_CCL18 [35, 36] and proinflammatory Mph_IL1B subset [37]. As expected, the M2 signature of macrophages decreased after BV while the M1 signature increased (Figure 4E; Figure S5G).

These activated DCs and macrophages initiate a cellular cascade that results in the activation of effector T cells. In responsive lesions, the average expression of effector genes, including *GZMB*, *GZMK* in CD8^+^ T cells increased while the nonresponsive lesions showed a more exhausted status with elevated *PDCD1*, *LAG3*, and *TIGIT* expression (Figure 4F), except for *IFNG*, which showed even higher expression in nonresponsive lesions. The chemotaxis of CD8^+^ T cells was enhanced after BV treatment in responsive lesions, which were strongly recruited by DCs (Figure 4G,H; Figure S5H). CD4^+^ Tm also showed enhanced activation in responsive lesions (Figure 4I), thereby mediating anti‐tumor immunity within the TME [38, 39]. They also showed enhanced responses to IFNα and IFNγ ligands in responsive lesions, which were blunted in nonresponsive lesions (Figure S5I).

Therefore, BV treatment‐induced TME remodeling in responsive lesions by enhancing the infiltration and anti‐tumor activation of effector cells, while nonresponsive lesions exhibited reduced proportions of effector cells and heightened CD8^+^ T cells exhaustion.

### 2.5 Distinct Resistance Mechanisms Emerge in CD30^+^ and CD30^−^ Malignant T Cells

BV‐induced ICD in malignant T cells, which subsequently activated anti‐tumor immunity in responsive lesions. Notably, while ICD was significantly decreased in CD30^−^ tumor cells from nonresponsive lesions, it remained largely unaltered in CD30^+^ tumor cells (Figure 2C). Based on these differential effects, we next investigated the specific mechanisms driving drug resistance in both CD30^+^ and CD30^−^ malignant T cell populations.

**FIGURE 5:**
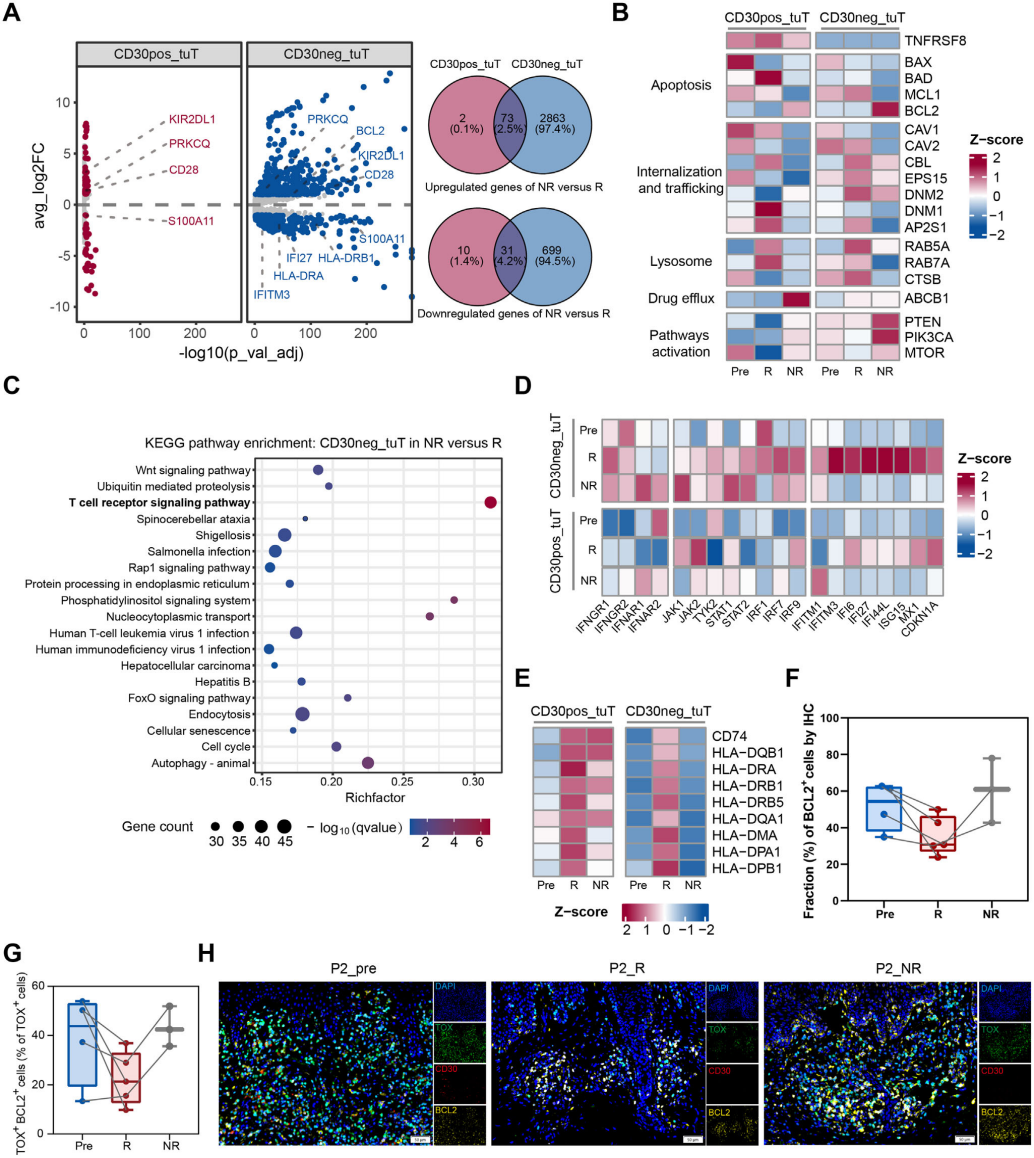
Molecular characteristics of malignant T cells in nonresponsive lesions. (A) Volcano plot (left) and Venn diagrams (right) showing the differentially expressed genes between CD30^+^ and CD30^−^ malignant T cells in NR and R lesions. (B) Heatmap of normalized expression of genes related to the action or resistant mechanisms of antibody‐drug conjugates in CD30^+^ and CD30^−^ malignant T cells from Pre, R, and NR groups. (C) KEGG enrichment analysis of the significantly expanded gene families in CD30^−^ malignant T cells in NR lesions compared to in R lesions. (D,E) Heatmap of normalized expression of IFNG and related genes (D), and *MHC‐II* genes (E) among CD30^+^ and CD30^−^ malignant T cells in Pre, R, and NR groups. (F) Boxplot showing the fraction of BCL2^+^ cells of each sample in Pre (*n* = 4), R (*n* = 5), NR (*n* = 3) groups by IHC staining. Paired samples were linked by gray lines. Two‐tailed paired Wilcoxon signed‐rank test was used. (G) Boxplot showing the fraction of TOX^+^BCL2^+^ cells within TOX^+^ malignant T cells of each sample based on multiplex IHC staining in Pre (*n* = 4), R (*n* = 5), NR (*n* = 3) groups. Paired samples were linked by gray lines. Two‐tailed paired Wilcoxon signed‐rank test was used. (H). Multiplex IHC staining of paired samples to determine the expression levels of TOX (green), CD30 (red), and BCL2 (yellow). DAPI (blue) was used to visualize cell nuclei. Scale bar = 50 µm.

We analyzed the differentially regulated genes of tumor cells between nonresponsive and responsive lesions, as well as the expression of genes reported or hypothesized to be involved in the action and/or resistance to ADCs [40]. Gene expression profiling revealed that the differentially regulated genes of CD30^+^ tumor cells between nonresponsive and responsive lesions and those of CD30^−^ tumor cells between the two types of lesions, shared an intersection subset while each also possessed a unique gene repertoire (Figure 5A; Table S5). Notably, CD30^+^ tumor cells harbored a unique upregulation of *ABCB1* (Figure 5B), which encodes a drug efflux transporter protein previously implicated in ADC resistance [41, 42]. Furthermore, these cells had impaired clathrin‐mediated endocytosis of BV and lysosome‐mediated release of MMAE (Figure 5B). In contrast, CD30^−^ tumor cells exhibited significantly enriched genes involved in the TCR signaling pathway (Figure 5C), and showed blunted responses to IFNα and IFNγ, characterized by decreased expression of interferon‐stimulated genes compared to those in responsive lesions (Figure 5D, 2J). Specifically, the MHC‐II molecules that were upregulated in responsive lesions were significantly downregulated in nonresponsive lesions (Figure 5E). Together with reduced ICD (Figure 2C), these defects prevent the presentation of antigens by tumor cells and weaken the activation of anti‐tumor cells.

However, a substantial overlap in transcriptional signatures was also noted. Both subsets showed increased expression of genes involved in the T cell activation/NF‐κB pathways (LAT, *PRKCQ*, *CARD11*, *CD28*, *CD6*). Notably, the expression of *BCL2*, encoding an antiapoptotic protein, was elevated in all malignant T cells from nonresponsive lesions, with the most pronounced upregulation observed in the CD30^−^ subset (Figure 5A,B). Consistently, whole‐section IHC staining of paired samples revealed a downward trend in BCL2 expression in responsive lesions and restoration in nonresponsive lesions (Figure 5F). However, BCL2 is also expressed by multiple nonmalignant immune populations within the tumor microenvironment, which may dilute tumor‐specific signals in whole‐section analyses [43]. By restricting the analysis to TOX^+^ malignant T cells, our multiplex IHC data revealed a decreased proportion of BCL2‐expressing malignant T cells in responsive lesions, with an opposite trend observed in nonresponsive lesions (Figure 5G,H). Together, these findings support a tumor cell‐intrinsic modulation of BCL2 associated with treatment response. Given that BCL2 has been reported to confer resistance to IFNγ‐induced apoptosis [44, 45], its upregulation may contribute to the impaired IFNγ response and consequent drug resistance in these tumor cells. Collectively, these data indicate that resistance to BV is mediated by similar yet distinct mechanisms in each subset, with CD30^−^ malignant T cells playing a predominant role.

### 2.6 Combination Therapy with BCL2 Inhibitors Overcomes BV Resistance

**FIGURE 6:**
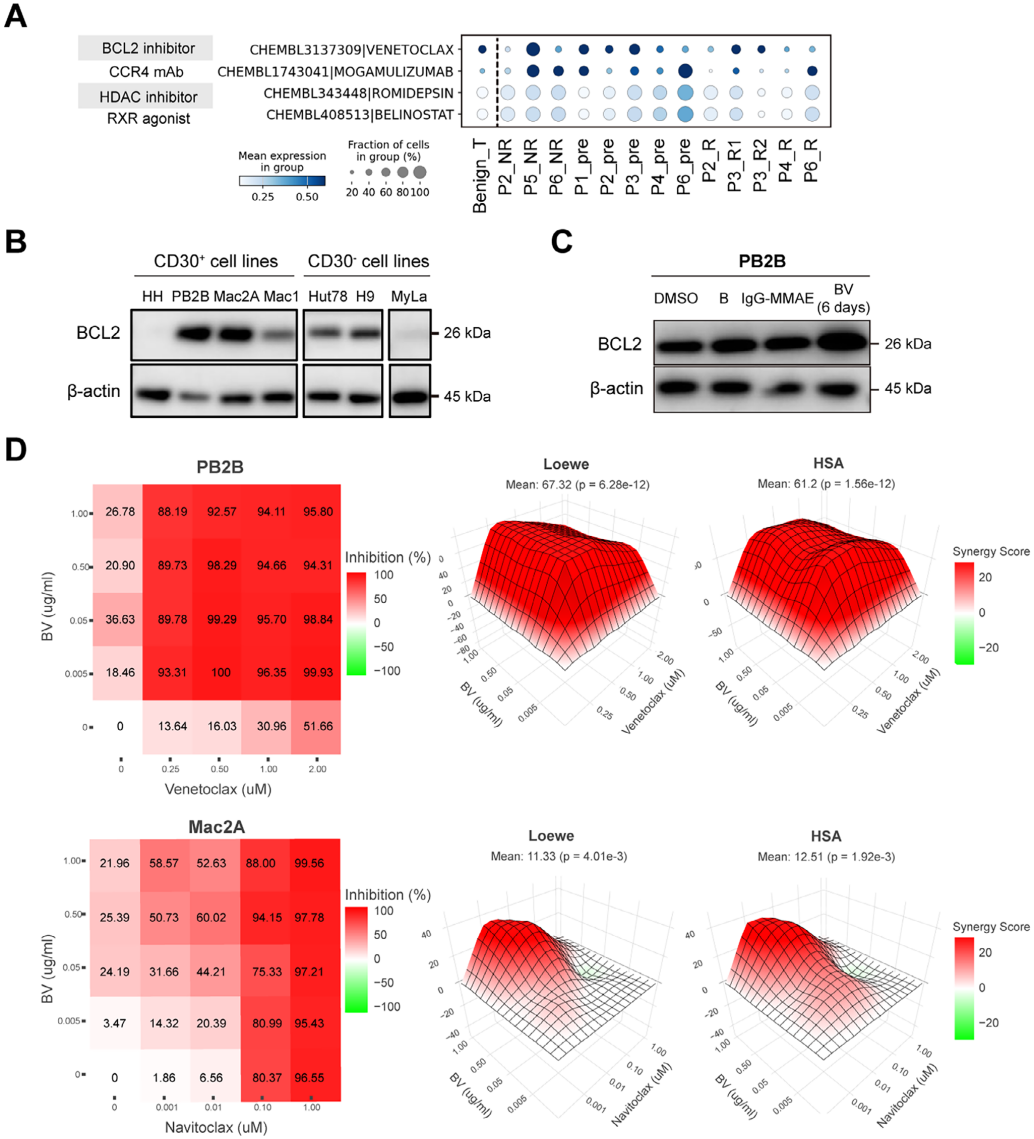
Synergistic effects of the combination of BV and BCL2 inhibitors in CTCL. (A) Dot plot showing the expression of drug targets predicted by drug2cell. (B) BCL2 protein expression of CTCL cell lines. (C) BCL2 protein expression of PB2B treated by BV and control agents for 6 days at 0.05ug/mL. (D) Percentage inhibition of PB2B (up) and Mac2A (bottom) with their synergy scores (Highest Single‐Agent (HSA) and Loewe models). The synergy score represents the magnitude of synergistic (>10), addictive (−10–10), or antagonistic effects (<−10).

To overcome the resistance to BV, drug2cell [46] was then performed on malignant T cells with benign CD4^+^ T cells as a comparator to evaluate drug target expression in single cells and therefore predict potential therapeutic targets. Several applicable drugs were identified for these patients, among them a highly selective BCL2 inhibitor, venetoclax, showed the highest score (Figure 6A). Venetoclax has shown promising effects in several lymphoma entities and been approved in treating patients with naïve and R/R chronic lymphocytic leukemia and acute myeloid leukemia [47, 48, 49]. We then investigated the combination effects of BV and BCL2 inhibitors in CTCL cell lines.

BCL2 expression was analyzed across all 7 CTCL lines (HH, PB2B, Mac2A, Mac1, Hut78, and H9), with the majority exhibiting high baseline expression, especially PB2B and Mac2A (Figure 6B). Furthermore, in drug‐resistant cells generated through continuous exposure to a low concentration of BV (0.05 µg/mL for 6 days), we confirmed a concomitant increase in BCL2 expression, suggesting its induction contributes to the resistant phenotype (Figure 6C).

Dose response analysis was conducted to investigate the combined effects of BV and two BCL2 inhibitors, venetoclax and navitoclax. Tumor cells were seeded and treated with BV or BCL2 inhibitors either as single agents or in combination, using a 5 × 5 concentration matrix. Cell viabilities were assessed and converted into Loewe and Highest Single‐Agent (HSA) scores as described in the method. A synergistic interaction was defined as a Loewe or HSA score exceeding 10 [50]. The inhibitory effect of BV on PB2B and Mac2A (high BCL2 expression levels) increased slightly with rising drug concentrations, while combination with venetoclax or navitoclax‐even at low concentrations‐promoted cell apoptosis with a statistically significant synergistic effect (Figure 6D; Figure S6A, and Table S6). Mac1 with lower BCL2 expression showed greater sensitivity to BV, and the combination with BCL2 inhibitor also enhanced the inhibitory effect when both drugs were used at low concentrations (Figure S6B). Collectively, we confirmed synergistic effects of BV in combination with BCL2 inhibitors across CTCL cell lines, highlighting that BCL2 inhibition can enhance sensitivity to BV in tumor cells.

## 3 Discussion

**FIGURE 7:**
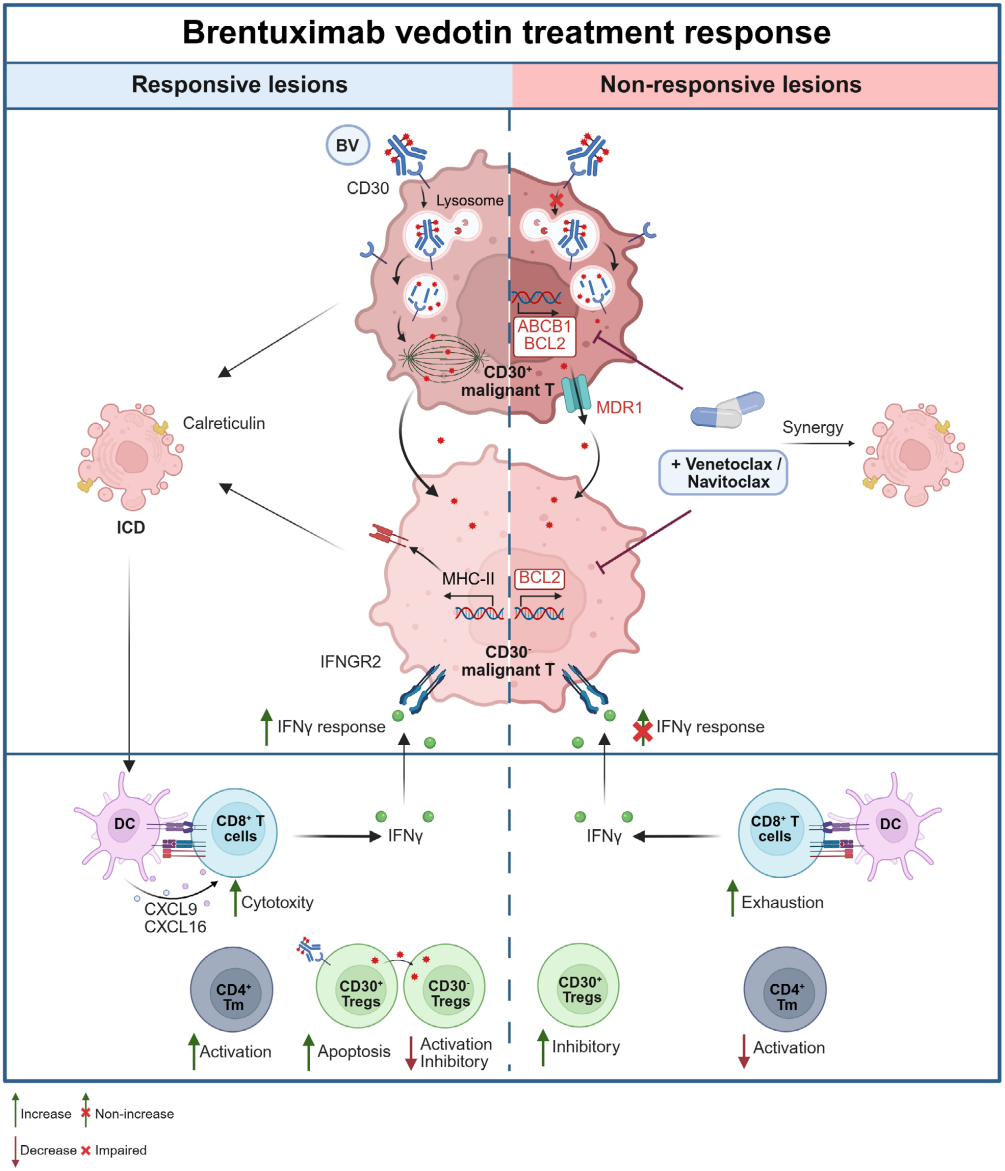
Summary of malignant T cells and TME dynamics in CD30^+^ MF during BV treatment.

The successful application of anti‐CD30 targeted therapy has advanced CTCL management, yet many patients experience persistent disease or progression. Current understanding of BV's mechanisms of action in CTCL primarily relies on preclinical studies and extrapolation from other CD30^+^ lymphomas, such as classic Hodgkin lymphoma (cHL) and anaplastic large cell lymphoma (ALCL). However, CD30 is heterogeneously expressed in CTCL, and the specific mechanisms governing response and resistance to BV remain poorly defined. Using scRNA‐seq on paired samples from CD30^+^ MF patients treated with BV, we depicted dynamic changes in tumor cells and the TME. Our analysis revealed that BV induces ICD in both CD30^+^ and CD30^−^ malignant T cells and augments IFNα and IFNγ signaling specifically in CD30^−^ cells. Furthermore, BV directly targeted TI‐Tregs, mitigating their immunosuppressive function and promoting anti‐tumor immunity. We identified distinct resistance mechanisms: CD30^+^ cells overexpressed the drug efflux transporter *ABCB1*, while CD30^−^ cells exhibited blunted IFNα and IFNγ responsiveness. Notably, our findings suggest that BCL2 inhibition may represent a promising strategy to overcome BV resistance in CTCL (Figure 7).

In responsive lesions (left), BV is internalized upon binding to CD30 on tumor cells. The payload drug MMAE is released from BV, diffuses into CD30^−^ tumor cells, and induces ICD in all tumor cells. ICD activates the anti‐tumor immunity mediated by DCs, CD8^+^ T, and CD4^+^ memory T cells. BV also targets CD30^+^ TI‐Tregs to suppresses their immunosuppressive activity. CD30^−^ tumor cells exhibit enhanced IFNγ response and elevated expression of MHC‐II molecules. In nonresponsive lesions (right), CD30^+^ tumor cells have impaired endocytosis of BV and upregulated expression of drug efflux gene *ABCB1*; CD30^−^ tumor cells exhibit blunted IFNγ response. All tumor cells have elevated expression of the antiapoptotic protein BCL2, and the combination of BV with BCL2 inhibitors shows significant synergistic effects. Created with BioRender.com.

CD30 is an activation‐associated antigen predominantly expressed on activated lymphoid cells and in various lymphoma entities [51, 52]. Previous studies have shown that it induces survival signals and promotes proliferation in T cell lymphomas through the activation of NF‐κB and MAPK signaling pathways [53, 54, 55]. Consistently, we observed that CD30^+^ malignant T cells exhibited higher levels of tumor markers and activation compared to their CD30^−^ counterparts. Previous studies have revealed that BV or free MMAE triggers ICD by inducing ER stress in CD30^+^ cHL, ALCL, and B cell lymphoma cell lines, which can further enhance the anti‐tumor activity within the TME [13, 56, 57]. Our study further revealed that the CD30^−^ tumor cells also underwent ICD in the presence of the CD30^+^ subset through bystander effect. This overcomes the heterogeneity in CD30 expression among tumor cells and augments the overall efficacy of BV [58, 59]. Moreover, clinical studies have confirmed that responses to BV are observed across a full range of CD30 expression levels [18, 60].

We further identified distinct transcriptional profiles associated with different pathologic responses of malignant T cells and the TME. A notable feature of responsive lesions is the enhanced responses to IFNα and IFNγ ligands and upregulation of MHC‐II molecule genes, especially in CD30^−^ malignant T cells. MHC‐II expression on tumor cells has been defined as an essential component of IFNγ‐related signatures and a predictor of the immunotherapy efficacy [28, 61, 62]. Studies have confirmed that optimal anti‐tumor responses occur when tumor cells express both MHC‐I and MHC‐II neoantigens [28, 63]. Collectively, MHC‐II‐mediated antigen presentation by tumor cells activates CD4^+^ T cells within the TME, which is a process required for efficient priming of MHC‐I restricted CD8^+^ T cells and their differentiation into functional cytotoxic T cells. Our study demonstrates that BV treatment significantly upregulates MHC‐II expression in CD30^−^ malignant T cells. This finding suggests that BV not only induces tumor cell death but also promotes anti‐tumor immunity by potentially enhancing antigen presentation. Moreover, multiple additional genes within the IFNγ signaling pathway were significantly upregulated in CD30^−^ tumor cells but not in the CD30^+^ subset. This indicates that beyond the direct elimination of CD30^+^ cells, a major effect of BV is the sensitization of CD30^−^ cells to enhanced anti‐tumor immune responses. The specific mechanisms through which BV selectively augments IFNγ signaling in the CD30^−^ tumor subset, however, remain to be clarified.

The remodeling of the TME involved the targeting of TI‐Tregs, which constitute another major direct target of BV due to their substantial CD30 expression. A previous study documented a reduction in TI‐Tregs within the peripheral blood of patients with relapsed Hodgkin lymphoma following BV treatment [57], with the mechanism unexplained. Our results now provide a mechanistic explanation for this BV‐mediated TI‐Tregs suppression. The selective targeting of TI‐Tregs to enhance anti‐tumor immunity, while minimizing autoimmunity, represents a promising strategy in cancer immunotherapy [64, 65]. Our findings contribute to the understanding of TI‐Tregs modulation and highlight its potential clinical implications. Concurrently, the DAMPs released from dying cells during ICD bind to specific pattern recognition receptors on DCs, strongly promoting DC maturation [25, 66]. In our patients, the antigen presentation capacity of DCs and the maturation of mregDCs were significantly enhanced in responsive lesions, which then could recruit and activate anti‐tumor cells, including cytotoxic CD8^+^ T cells to launch effective immune response toward malignant T cells [13, 57].

Our investigation into the mechanisms of BV nonresponsiveness revealed that intrinsic features of malignant T cells are primary contributors. CD30^+^ malignant T cells showed impaired clathrin‐mediated endocytosis of BV, with upregulated drug efflux gene *ABCB1*, consistent with previous reports of *ABCB1* overexpression in BV‐resistant cHL cell lines [42, 67]. In contrast, CD30^−^ tumor cells exhibited reduced expression of interferon‐stimulated genes, including MHC‐II molecules, rendering them nonresponsive to the anti‐tumor immune microenvironment. Noteworthy, these cells showed significant overexpression of the anti‐apoptotic protein BCL2. Previous studies have identified BCL2 downregulation as a marker for efficient apoptosis induced by BV and MMAE‐conjugated ADCs in non‐Hodgkin lymphoma cell lines [68, 69]. In this study, we observed its upregulation in nonresponsive lesions, and demonstrated induction of BCL2 during acquired BV resistance in vitro. BCL2 inhibition has been shown to effectively induce apoptosis in CTCL patient‐derived malignant cells, and its combination with HDAC inhibition or NF‐κB inhibitors yields synergistic killing of CTCL tumor cells [70, 71]. We further confirmed a promising synergistic effect between BV and two BCL2 inhibitors across CTCL tumor cell lines. Moreover, BCL2 has been reported to be involved in resistance to IFNγ‐induced apoptosis, which may contribute to the impaired IFN responses we observed [44, 45]. Collectively, the combination of BV with BCL2 inhibitors represents a practical strategy to overcome resistance to BV, warranting further validation.

## 4 Conclusion

In summary, BV exerts a dual mechanism in mycosis fungoides by targeting malignant T cells and remodels the immunosuppressive microenvironment. We identify distinct resistance pathways and demonstrate that combining BV with BCL2 inhibitors overcomes resistance, offering a promising strategy to improve the clinical management of late‐stage MF, which currently does not have a cure.

## 5 Methods

### 5.1 Patient Samples

CD30^+^ MF patients (*n* = 6) who failed prior systemic treatment and received BV monotherapy (1.8 mg/kg, q3w for 16 cycles) at the Skin Lymphoma Clinic of Peking University First Hospital in the period from January 2023 to January 2025 were enrolled. All diagnoses were verified by at least two dermatopathologists according to previously described criteria [72]. For scRNA‐seq, thick plaques or tumors were selected and resected from 6 patients yielding 13 samples, including 5 pretreatment lesions and 8 posttreatment samples at points consistent with reported time to response [16, 17]. Each freshly dissociated sample was transported to the laboratory immediately. Samples posttreatment were categorized into two groups based on pathological response: major pathologic responsive (R, *n* = 5) and nonresponsive lesion (NR, *n* = 3). All participants provided written consent for specimen collection and analysis under the study protocol approved by the Peking University First Hospital Ethics Committee (No. 2020Y005). The clinical information of all patients was summarized in Table S1.

### 5.2 Single Cell Preparation and Sequencing

Fresh tissues were dissociated into single‐cell suspensions using gentleMACS (Miltenyi Biotec, 130‐095‐937) and processed on the 10× Genomics Chromium platform for scRNA‐seq and TCR αβ sequencing following the manufacturer's instructions. Transcriptome and TCR library reads were aligned to GRCh38 and ‘refdata‐cellranger‐vdj‐GRCh38‐alts‐ensembl‐7.1.0’ using Cell Ranger (v.7.2.0) and CellRanger vdj (v.7.2.0), respectively.

### 5.3 Quality Control and Clustering

After quality control (filtering cells with <500 or >6,000 genes, >10% mitochondrial reads) and removal of doublets by DoubletFinder (version 2.0.3), 97 044 cells were retained. Data integration was performed by Harmony (version 1.2.0) [73]. Dimension reduction, cell clustering, and differential gene expression were performed by Seurat (version 4.0.5). The resolution parameters of the FindClusters function were different for different cell types, with 0.6 for all cells, 0.6 for T and NK cells, and 0.5 for myeloid cells. Major cell lineages were assigned to each cluster of cells using the abundance of canonical marker genes using the FindAllMarkers function with the parameter “min.pct = 0.25, logfc.threshold = 0.25”. Clusters expressing two or more major lineage markers on UMAP plots were identified as doublets and excluded.

### 5.4 Inferring CNVs and TCR Clonotype Analysis

To identify malignant T cells, CNVs were inferred from the scRNA‐seq data using the R infercnv package (version 1.10.1). NK and CD8^+^ T cells were used as controls for the CNV analysis. We estimated the CNV patterns of all cells in each sample using default parameters as described previously [74]. Clonotype frequency and CDR3 amino acid sequences were incorporated into the metadata slot of the Seurat objects by the Seurat package. As previously described, malignant T cells from samples of the same patient harbored the same unique TCR clonotype [74]. Malignant clone was designated as the most abundant clonotype in each patient respectively. Other clonotypes with lower frequencies were grouped as “nonmalignant clone”, while a subset of T cells lacked detectable TCR clonotype assignments. Collectively, malignant T cells were defined by CNVs and matched TCRα and TCRβ clonotypes at the single‐cell level.

### 5.5 Enrichment Analysis and Signature Scores

Differential expression analysis comparing cells from treatment exposure or response groups was performed using the FindMarkers function with the parameter “min.pct = 0.1, logfc.threshold = 0.1”. Genes with avg_log2FC > 0.25 or < −0.25 and adjusted *p* value < 0.05 were identified as up‐ or downregulated genes for further functional enrichment analysis. The fgsea, GSVA R packages (v.1.20.0 and v.1.42.0) were used to calculate enrichment scores, and the Molecular Signature Database (MSigDB), GO terms, and KEGG pathways with the most significant *p* values were shown.

We calculated the G2M scores of each cell with its prediction classification in G2M phase based on 54 G2/M genes [75] by the CellCycleScoring R function. To evaluate the signatures of malignant T cells and immune cells, the signature scores were calculated by AddModuleScore function in Seurat. The gene lists and corresponding references were listed in Table S7. The average score of each sample was calculated for clusters containing ≥ 7 cells.

### 5.6 Cellular Fraction Calculation

We calculated the cellular fractions of major cell types and clusters in TME and their major cell types in each sample, respectively. Samples with fewer than seven cells in a major cell type were excluded. The proportion of CD30^+^ cells within their respective clusters was calculated. The differences among response groups for the fractions was compared using two‐tailed unpaired Wilcoxon rank‐sum test.

### 5.7 Pseudotime Analysis

Monocle2 (version 2.22.0) and CytoTRACE (Cellular Trajectory Reconstruction Analysis using gene Counts and Expression, version 0.3.3) were used to infer the cell differentiation trajectories of DCs. The expression matrices of the scRNA‐seq data were analyzed with default settings following the vignette of Monocle 2. CytoTRACE was then utilized to determine the recommended time order of cells as described [76]. CytoTRACE scores of subtypes of DCs were calculated and ranged from 0 to 1, while higher scores indicate higher stemness (less differentiation) and vice versa.

### 5.8 Cell–Cell Interaction Analysis

CellPhoneDB (version 3.0.0) was used to infer cell–cell interaction between different cell clusters based on gene expression level [32]. The significant cell–cell interactions were selected with a *p* value threshold of <0.05. We used the R ggplot2 (version 3.5.1) package to visualize the results. NicheNet (version 2.0.4) is a powerful tool that predicts cellular intercommunication and ligands driving the transcriptomic changes of target cells [77]. In our study, the receiver cell population is the CD30^+^ or CD30^−^ malignant T cells, whereas the other immune cells were sender cell populations. We identified potential ligands that drive the unique phenotype of CD30^+^ and CD30^−^ malignant T cells after treatment in response as described previously [30].

### 5.9 Reagents and Confirmation of Combination Strategies

BV (HY‐P99107A), brentuximab (the CD30‐directed monoclonal antibody part of BV, HY‐P99151) were purchased from MedChemExpress (Monmouth Junction, NJ, USA). IgG‐MMAE (HY‐X4805), a nonbinding ADC, was synthesized by MedChemExpress. It has the same components as BV, whereas the monoclonal antibody targeting CD30 is replaced by human IgG1 kappa (HY‐P99001) that does not bind to the target. BCL2 inhibitors, including venetoclax (HY‐15531) and navitoclax (HY‐10087) were also purchased from MedChemExpress.

Drug2cell was performed on malignant T cells with benign CD4^+^ T cells as a comparator to predict potential druggable targets [78]. For synergy analysis, CD30^+^ CTCL cell lines (Mac1, Mac2A, PB2B, HH) were seeded in 96‐well plates at a density of 5 × 10^4^/mL, and treated with BV or one of the BCL2 inhibitors (venetoclax or navitoclax), either as single agents or in combination, using a 5 × 5 concentration matrix for 24 or 48 h. The concentration ranges were 0–1 ug/ml for BV, 0–2 µm for venetoclax, and 0–5 µm for navitoclax. Cell viabilities were assessed by CCK‐8 assay (C0038, Beyotime), and the anti‐tumor effects were analyzed by SynergyFinder 3.0 (https://synergyfinder.org/) under Loewe and HSA models [50], with the synergy score >10 indicating a synergistic effect.

### 5.10 Immunohistochemistry and Multiplex Immunohistochemistry

Paraffin‐embedded tissue sections were dewaxed and rehydrated. After antigen retrieval, endogenous peroxidase inactivation and blocking, slides were incubated with following antibodies: CD30 (MAB0023, MXB Biotechnologies); CD8A (ab17147, Abcam); CD11c (ab52632, Abcam); BCL2 (RMA0660, MXB Biotechnologies). IHC slides were scanned by a NanoZoomer microscopic slide scanner (Hamamatsu Photonics, Japan) and visualized by NDP.View2 software. Quantification of the fractions of positive cells from the IHC images was quantified by a professional pathologist. To evaluate the alterations in specific CD30^+^ and CD30^−^ malignant T cells, TI‐Tregs, and BCL2 expression in malignant T cells, we performed multicolor IHC saining on paraffin‐embedded tissue sections of all patients in our cohort. The PANO 7‐plex IHC Kit (cat0004100100, Panovue, Beijing, China) was used with the following primary antibodies: TOX (1:200, ab237009, Abcam), FOXP3 (1:200, BX50188, Biolynx), CD30 (1:200, 54535S, Cell Signaling Technology), and BCL2 (1:400, ab182858, Abcam). Primary antibodies were applied sequentially, followed by secondary antibody incubation and tyramide signal amplification (TSA). Antigen retrieval with heat‐treatment was performed on the slides between each round. Finally, nuclei were stained with 4′‐6′‐diamidino‐2‐phenylindole (DAPI, SIGMA‐ALDRICH). Slides were scanned by Olympus VS200 (Olympus Germany) with Olympus UPLXAPO 20× objective lens. Quantification of the density or fraction of positive cells was performed using ImageJ software and a professional pathologist. Differences between paired samples were compared using two‐tailed paired Wilcoxon signed‐rank tests.

### 5.11 Cell Death Analysis

Apoptosis was measured using a detection method based on Annexin V, following the manufacturer's instructions. Furthermore, the composition of the cell cycle was analyzed through propidium iodide (PI)‐mediated flow cytometry (BD Biosciences). ICD was measured by the cell surface calreticulin (19780S, CST). Flow cytometry data were processed with FlowJo and ModFit software (BD Biosciences).

### 5.12 Statistical Analysis

Statistical analyses and data visualization were performed using R software (version 4.1.0) or GraphPad Prism 8 (GraphPad Software, San Diego, CA, USA). Differences in signature scores between different groups were assessed using two‐tailed unpaired Wilcoxon rank‐sum test for scRNA‐seq data. For in vitro experiments, statistical significance was assessed using two‐tailed unpaired *t*‐tests or one‐way ANOVA followed by Dunnett's post‐hoc test, as appropriate. For paired samples obtain from the same patients before (Pre) and after treatment (R), two‐tailed paired Wilcoxon signed‐rank tests were applied. A total of 13 samples (Pre: *n* = 5, R: *n* = 5, NR: *n* = 3) were included in the scRNA‐seq and CD30 IHC analyses, while 12 samples (Pre: *n* = 4, R: *n* = 5; NR, *n* = 3) were included for other IHC markers (CD8A, CD11c, BCL2) and multiplex IHC analyses. Owing to the limited number of matched samples (*n* = 4), formal statistical significance could not be achieved. Therefore, changes were additionally evaluated based on the consistency and directionality of patient‐level responses. Statistical significance was set at *p* or adjusted *p* value < 0.05. Detailed materials and methods are provided in Supporting Information.

## Author Contributions

YW conceived the study, directed and supervised research. YW and JRS reviewed the histopathologic diagnoses. YW, JRS and YJ participated in the preparation of clinical samples. YJ, PL, and MJL gathered detailed clinical information. YJ, PL, and MJL conducted the bioinformatics investigation. YJ, YJW, MZC, YX, and ZJC conducted experiments and interpreted the data. All the authors contributed to manuscript preparation. YJ wrote the original manuscript. JRS and YW reviewed and edited the manuscript. SJR and YW are the corresponding authors of this manuscript.

## Funding

This study was supported by the Capital's Funds for Health Improvement and Research (Grant No. 2024‐1‐4074); the Beijing Natural Science Foundation (Grant No. Z240016); and the National Natural Science Foundation (Grant No. 82425050); the National Natural Science Foundation of China (Grant No. 82002903, J.S.).

## Conflicts of Interest

The authors declare no conflicts of interest.

## Supporting information




**Supporting File 1**: advs74582‐sup‐0001‐SuppMat.docx.

**Supporting File 2**: advs74582‐sup‐0002‐TableS1.xlsx.

**Supporting File 3**: advs74582‐sup‐0003‐TableS2.xlsx.

**Supporting File 4**: advs74582‐sup‐0004‐TableS3.xlsx.

**Supporting File 5**: advs74582‐sup‐0005‐TableS4.xlsx.

**Supporting File 6**: advs74582‐sup‐0006‐TableS5.xlsx.

**Supporting File 7**: advs74582‐sup‐0007‐TableS6.xlsx.

**Supporting File 8**: advs74582‐sup‐0008‐TableS7.xlsx.

## Data Availability

All data are available in the main text or in supplemental material. The scRNA‐seq data were deposited in the Gene Expression Omnibus database under accession numbers GSE303446. For original data, please contact Yang Wang (yangwang_dr@bjmu.edu.cn).
