# Supplementary material for: Beyond CD30: Dual‐Targeting of Malignant and Regulatory T Cells by Brentuximab Vedotin Remodels the Lymphoma Microenvironment and Overcomes Resistance via BCL2 Inhibition in Mycosis Fungoides
Source: Adv Sci (Weinh). 2026 Feb 28;13(26):e17353. doi: 10.1002/advs.202517353 (PMC13159111; doi:10.1002/advs.202517353)
Supplement: Supplementary file 1 — Supporting File 1: advs74582‐sup‐0001‐SuppMat.docx. [file ADVS-13-e17353-s001.docx]

**Supplementary Information**

## **Supplementary materials and methods**

### **Sample Processing**

Fresh samples were transported rapidly to the laboratory, cut into pieces (1-3 mm^3^) on ice and transferred into gentleMACS C tubes (Miltenyi Biotec, 130-093-237). Tissues were digested in an enzyme mix  (130-095-929, Miltenyi Biotec) on a gentleMACS octo Dissociator (Miltenyi Biotec, 130-095-937). Cell suspensions were filtered by 70-μm strainers (Corning, 352350). Cell debris was excluded by Debris Removal Solution (130-109-398, Miltenyi Biotec) according to the manufacturer’s instructions. Red blood cells (RBC) were then removed using RBC lysis buffer (C3702, Beyotime). The cell suspension was loaded onto a Chromium single-cell controller (10X Genomics) to generate single-cell gel beads in the emulsion in accordance with the manufacturer’s instructions. Gene expression libraries were generated from the resulting complementary DNA after clean-up following the 10x Genomics protocols. TCR αβ sequences were also enriched from the cDNA and following the instructions of the V(D)J Kit workflow by 10X Genomics with no adjustments.

### **Sample integration and clustering**

After discarding poor-quality cells, a total of 97,044 cells were retained for downstream analysis. The gene expression matrices were normalized by the NormalizeData function using scale.factor =10,000, and 3000 features with high cell-to-cell variation were calculated using the FindVariableFeatures function. To adjust for biological sources of variation between samples, we used the “RunHarmony” function in harmony package (version 1.2.0) for dataset integration^1^. Principal components analysis (PCA) was performed on the integration-transformed expression matrix using the RunPCA function, and the first 20 principal components (PCs) were used in the FindNeighbors function. The resolution parameters of the FindClusters function were different for different cell types, with 0.6 for all cells, 0.6 for T and NK cells, and 0.5 for myeloid cells. The original log-normalized expression values were used for all differential expressions and gene set level analyses. Uniform manifold approximation and projection (UMAP) was performed for visualization in two dimensions using the RunUMAP function with the 20 PCs and other default parameters. Major cell lineages were assigned to each cluster of cells using the abundance of canonical marker genes, and marker genes for each cluster were found using the FindAllMarkers function with the parameter “min.pct = 0.25, logfc.threshold = 0.25”. We also manually filtered the clusters that expressed two or more major lineage markers on UMAP plot and probably were doublets that were not recognized by DoubletFinder.

### **NicheNet analysis**

NicheNet (version 2.0.4) is a powerful tool that predicts cellular intercommunication and ligands driving the transcriptomic changes of target cells ^2^. It uses human or mouse scRNA-seq datasets as inputs in combination with a database of known ligand-to-target signaling paths to infer possible interactions between different cell types. NicheNet prioritizes ligands according to their activity and looks for affected targets with high potential to be regulated by these prioritized ligands. In our study, the receiver cell population is the CD30^+^ or CD30^-^ malignant T cells, whereas the other immune cells were sender cell populations. The gene sets of interest were differentially expressed genes (DEGs) in CD30^+^ or CD30^-^ malignant T cells in R compared to Pre group.

In addition, we also identified potential ligands that drive the unique phenotype of CD30^+^ and CD30^-^ malignant T cells after treatment in R as described previously^3^. The top 50 genes of DEGs ordered by log2FC with log2FC > 0.5 and adjusted *P* value < 0.05 were used as gene set of interest, while all expressed genes of CD30^-^ malignant T cells were used as the background of genes. Genes were considered as expressed when they have nonzero values in at least 10% of the cells in a cell type. We only used the expressed receptors in CD30^-^ malignant T cells to construct the expressed ligand-receptor interactions and calculate the ligand activity.

### **Prediction of druggable targets using drug2cell**

To predict potential druggable targets on malignant T cells, we ran drug2cell ^4^ on malignant T cells together with benign CD4^+^ T cells as a comparator. Drug2cell is a druggable target prediction tool that integrates drug–target interactions from the ChEMBL database (https://www.ebi.ac.uk/chembl) with single-cell data to comprehensively evaluate drug target expression in single cells. We first calculated per-cell scores of ChEMBL drug targets using the d2c.score() function. Then, we performed differentially expressed analysis on ChEMBL drugs by comparing normal CD4^+^ T cells and malignant T cells using the scanpy tl.rank_genes_groups() function. When visualizing the result, we separated malignant T cells by samples to show drugs that potentially function in multiple in each sample, given the strong inter-patient heterogeneity of CTCL tumors.

### **Cell lines**

Human CTCL cell lines Mac1, Mac2A, PB2B, HH, Hut78, H9, MyLa were cultured in RPMI-1640 with 10% fetal bovine serum containing 100 U/mL penicillin and 0.1 mg/mL streptomycin. Human CTCL cell line MJ was cultured in IMDM with 20% fetal bovine serum, 100 U/ml penicillin, and 0.1 mg/ml streptomycin. Peripheral blood mononuclear cells (PBMCs) were obtained from 2 healthy donors by Ficoll-Paque PLUS (GE Healthcare). Peripheral CD4^+^ T cells were purified from PBMCs using a CD4^+^ T cell isolation kit (480009, Biolegend).

### **Flow cytometry**

For the expression of CD30, cells were collected and incubated with mouse anti-human CD30 antibody (333906, Biolegend), or isotype-matched control antibody (HY-P99001, MedChemExpress) for 15 min at 4 °C in dark. After washing with phosphate-buffered saline (PBS), data were required by ﬂow cytometry and the proportions of CD30 positive cells were quantified by FlowJo.

### **Western blotting analysis**

Whole cell lysates were prepared with Total Protein Extraction Kit (KGP250, KeyGEN). Protein concentration was measured using Pierce BCA Protein Assay Kit (23227, Thermo Fisher). 4-20% gradient SDS-polyacrylamide electrophoresis and immunoblot analyses were performed. Antibodies were shown as follows: CD30 (54535S, Cell Signaling Technology), GAPDH (5174S, Cell Signaling Technology), BCL2 (ab182858, Abcam), β-actin (4970S, Cell Signaling Technology), ProteinFind^®^ Goat Anti-Rabbit IgG (H+L), HRP Conjugate (HS101-01, TransGen Biotech), ProteinFind^®^ Goat Anti-Mouse IgG (H+L), HRP Conjugate (HS201-01, TransGen Biotech).

### **Treatment groups of brentuximab vedotin**

For apoptosis analysis, CTCL cell lines were seeded in 24-well plates at a density of 1×10^5^/ml, treated with dimethyl sulfoxide (DMSO), venetoclax (0.2, 1 or 4 μM) , or navitoclax (0.001, 0.01, 0.1, 1 or 5μM) for 24 hours. CTCL cell lines were also treated with 0.5 or 1 μg/ml of brentuximab vedotin (BV), as well as controls of brentuximab, IgG-MMAE, and DMSO at the same concentrations for 24 or 48 hours. The specific concentrations and durations of drug usage were all labeled in the figures.

**References:**

1. Korsunsky I, Millard N, Fan J, et al. Fast, sensitive and accurate integration of single-cell data with Harmony. *Nat Methods* 2019;16(12):1289-96. doi: 10.1038/s41592-019-0619-0 [published Online First: 2019/11/20]

2. Browaeys R, Saelens W, Saeys Y. NicheNet: modeling intercellular communication by linking ligands to target genes. *Nat Methods* 2020;17(2):159-62. doi: 10.1038/s41592-019-0667-5 [published Online First: 2019/12/11]

3. Cheng S, Li Z, Gao R, et al. A pan-cancer single-cell transcriptional atlas of tumor infiltrating myeloid cells. *Cell* 2021;184(3):792-809.e23. doi: 10.1016/j.cell.2021.01.010 [published Online First: 2021/02/06]

4. Kanemaru K, Cranley J, Muraro D, et al. Spatially resolved multiomics of human cardiac niches. *Nature* 2023;619(7971):801-10. doi: 10.1038/s41586-023-06311-1 [published Online First: 2023/07/13]

## **Supplementary tables**

**Table S1.** Clinical characteristics of patients.

**Table S2.** Differentially expressed genes of malignant T cells compared to normal CD4^+^ T cells (Tregs excluded).

**Table S3.** Differentially expressed genes of CD30^+^ malignant T cells compared to CD30^-^ malignant T cells pre-treatment.

**Table S4.** Differentially expressed genes of T/NK clusters in responsive lesions compared to pre-treatment lesions.

**Table S5.** Differentially expressed genes of CD30^+^ and CD30^-^ malignant T cells in non-response compared to responsive lesions, respectively.

**Table S6.** Synergy scores of all combinations of CD30^+^ CTCL cell lines.

**Table S7.** Gene list of signature scores.

## **Supplementary figures**


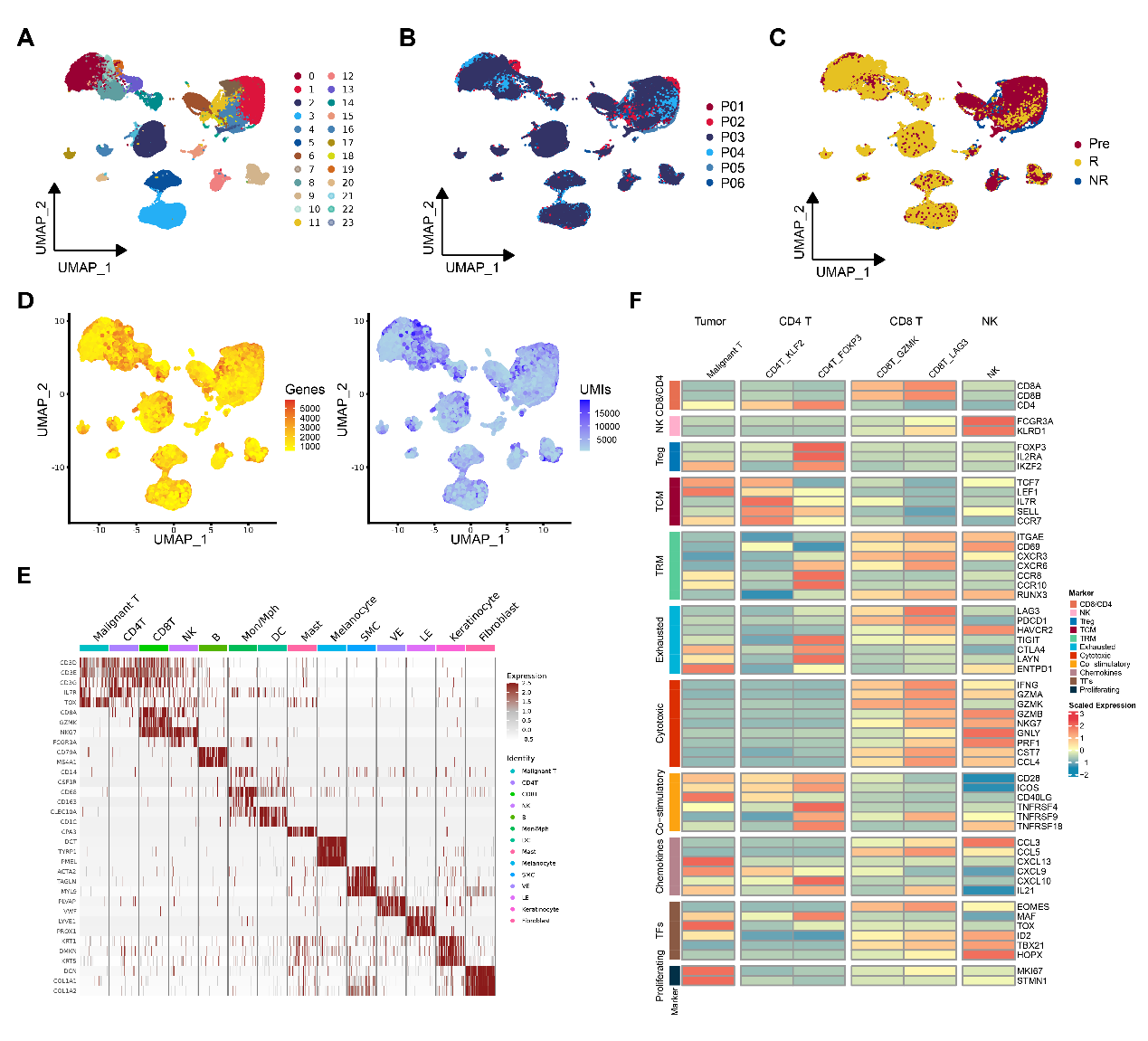


**Figure S1. Single-cell clustering and marker gene visualization for major cell types and T/NK clusters.**

(A). UMAP plot of all cells categorized into 24 clusters. (B, C). UMAP plots showing no batch effects from patients (B) and treatment (C) groups. (D). UMAP plots showing the number of detected genes and unique molecular identifiers for all cells. (E, F). Heatmap showing the expression of selected marker genes for the major cell types (E) and T/NK clusters (F).


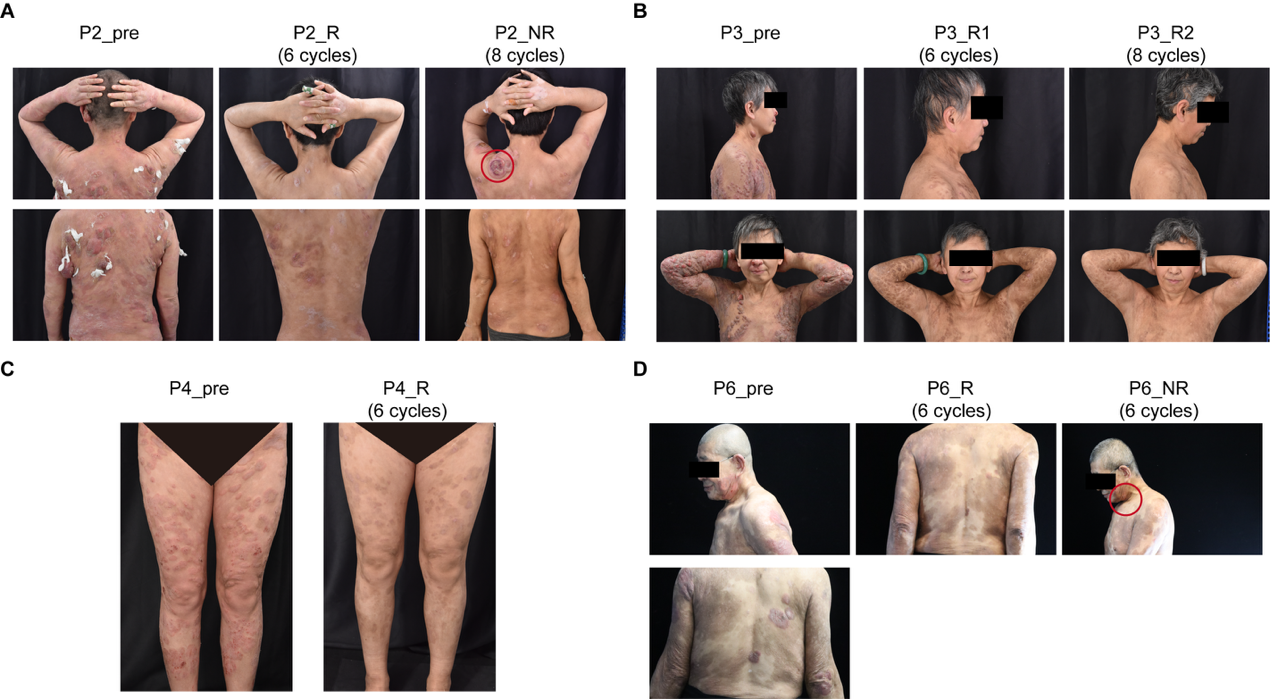


**Figure S2. Representative clinical images of paired samples from four patients before and after treatment.**

Pre- (labeled as “_pre”) and post-treatment images show significant improvement in responsive lesions (“_R”) from all patients. Newly developed (P2_NR) and persistent (P6_NR) lesions during treatment are marked with red circles.


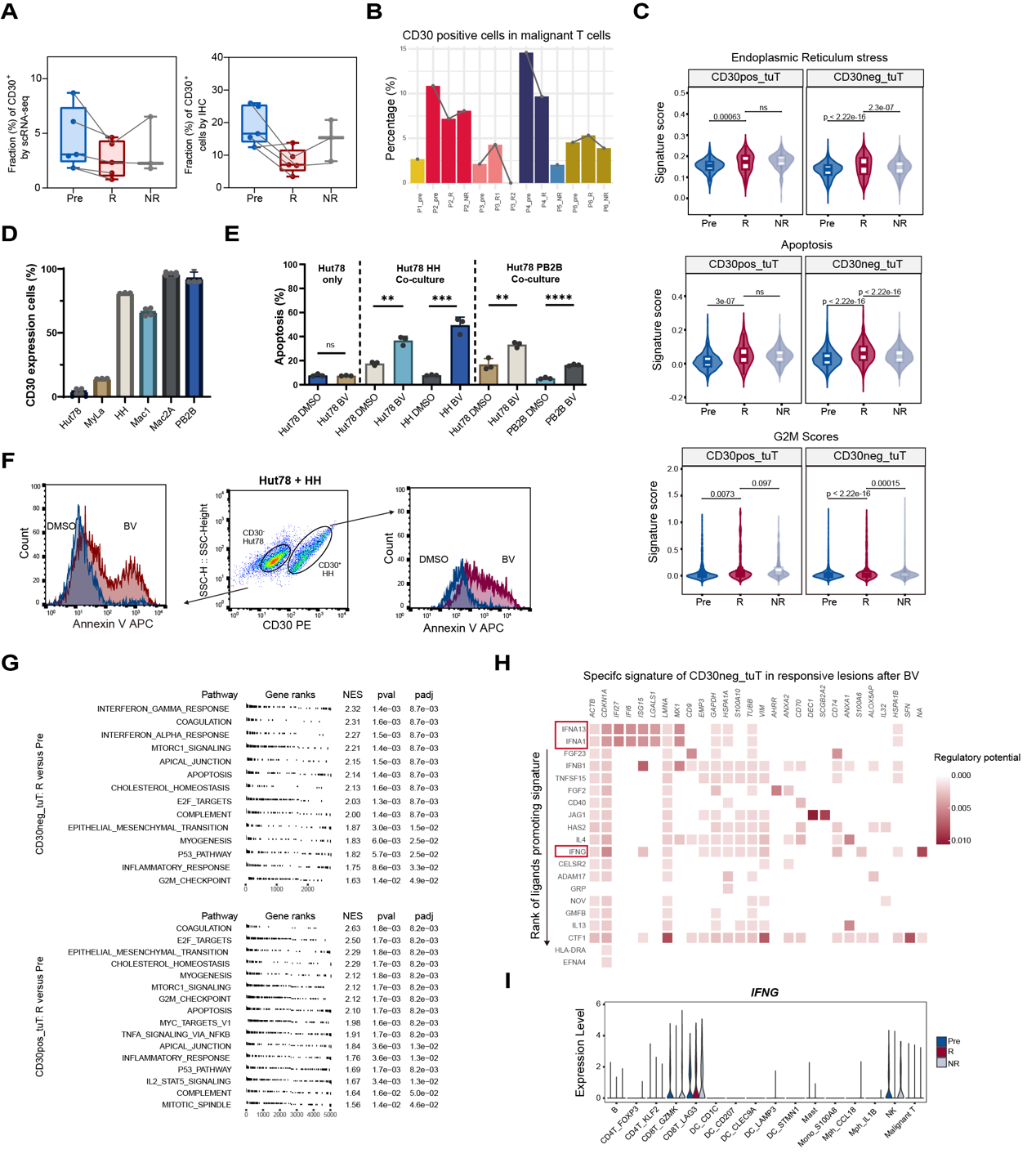


**Figure S3. Post-treatment remodeling of malignant T cells in responsive lesions.**

(A). Boxplot showing the fraction of CD30^+^ cells of each sample based on scRNA-seq (left) and IHC staining (right) in Pre (n = 5), R (n = 5), NR (n = 3) groups. Paired samples were linked with gray lines. Two-tailed paired Wilcoxon signed-rank test was used. (B). Bar plot showing the fraction of CD30^+^ cells of malignant T cells from each sample based on mRNA expression levels. Paired samples were linked with gray lines. (C). Violin plots showing signature scores of ER stress, apoptosis and G2M scores for CD30^+^ and CD30^-^ malignant T cells in Pre, R, and NR groups, respectively. Two-tailed unpaired Wilcoxon rank-sum test was used. (D). The percentage of CD30 expression in CTCL cell lines evaluated by flow cytometry analysis. (E). Bar plots showing apoptosis rates of CD30^+^ tumor cells (PB2B or HH), and CD30^-^ Hut78 under mono- and co-culture conditions. Unpaired t test was used. (F). Flow cytometry analysis of apoptosis of CD30^+^ HH and CD30^-^ Hut78 under mono- and co-culture conditions. (G). Significantly enriched pathways of CD30^+^ (bottom) and CD30^-^ (up) malignant T cells in R lesions compared to Pre lesions by GSEA analysis, respectively. (H). Heatmap showing potential ligands driving the phenotype of CD30^-^ malignant T cells in responsive lesions, IFN ligands were enclosed by red box. (I). Violin plots showing the expression of IFNγ genes of cell clusters in Pre, R and NR groups.


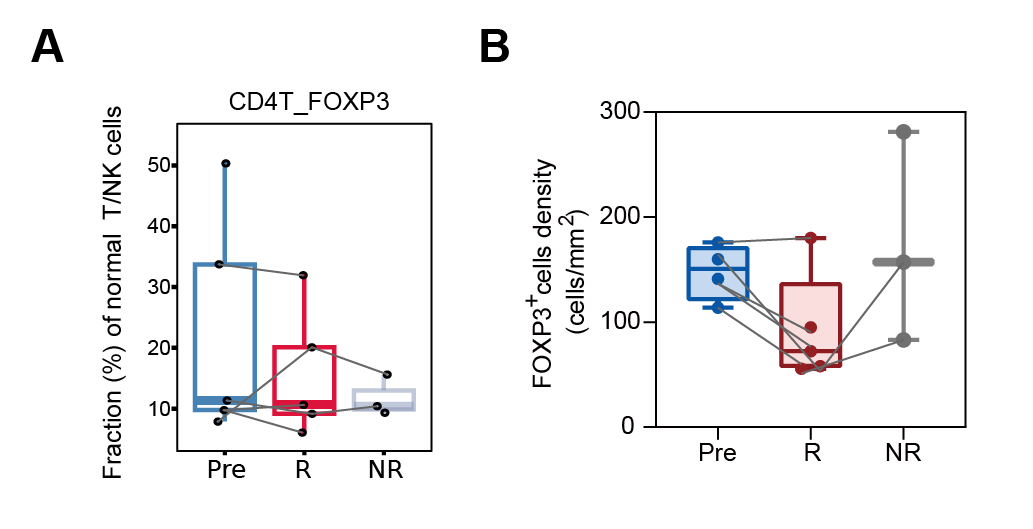


**Figure S4. TI-Tregs as additional target of BV with decreased proportions after treatment.**

(A). Boxplots showing cellular fractions of TI-Tregs within normal T/NK cells in Pre (n = 5), R (n = 5), NR (n = 3) groups. Center line indicates the median value, lower and upper hinges represent the 25th and 75th percentiles, respectively, and whiskers denote 1.5× interquartile range. Each dot corresponds to one sample. Paired samples were linked gray lines. Two-tailed paired Wilcoxon signed-rank test was used. (B). Boxplot showing the cell density of FOXP3^+^ TI-Tregs of each sample based on multiplex IHC staining in Pre (n = 4), R (n = 5), NR (n = 3) groups. Paired samples were linked with gray lines. Two-tailed paired Wilcoxon signed-rank test was used.


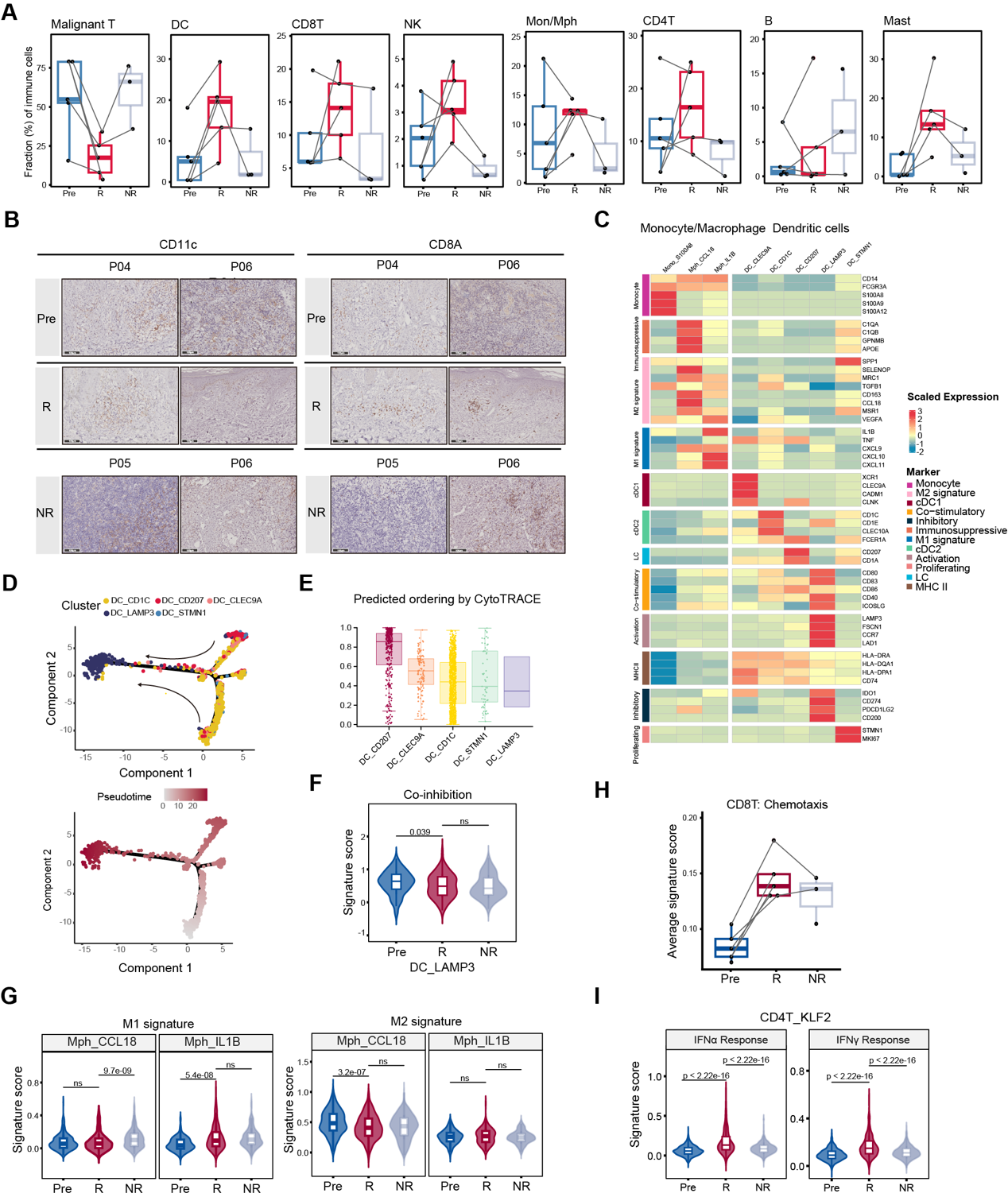


**Figure S5. Reprogramed tumor microenvironment support antitumor immunity after BV treatment.**

(A). Boxplots showing cellular fractions of major cell types of TME in Pre (n = 5), R (n = 5), NR (n = 3) groups. Center line indicates the median value, lower and upper hinges represent the 25th and 75th percentiles, respectively, and whiskers denote 1.5× interquartile range. Each dot corresponds to one sample. Paired samples were linked by gray lines. Two-tailed paired Wilcoxon signed-rank test was used. (B). IHC staining (200×) of DCs (CD11c) and CD8^+^ T (CD8A) in Pre, R and NR lesions from another three patients, respectively. (C). Heatmap showing the expression of selected marker genes for the clusters of myeloid cells. (D). The developmental trajectory of DCs clusters (top) and pseudotime (bottom) inferred by Monocle2. (E). Box plot of CytoTRACE scores evaluating the stemness of five clusters of DCs. (F). Violin plots showing signature scores of co-inhibition effect of mregDCs (DC_LAMP3) in Pre, R, and NR groups. Two-tailed unpaired Wilcoxon rank-sum test was used. (G). Violin plots showing signature scores of M1 and M2 of macrophage clusters in Pre, R, and NR groups. Two-tailed unpaired Wilcoxon rank-sum test was used. (H). Boxplot of the average signature score of chemotaxis in CD8^+^ T cells in Pre (n = 5), R (n = 5), NR (n = 3) groups. Paired samples were linked gray lines. Two-tailed paired Wilcoxon signed-rank test was used. (I). Violin plots showing signature scores of IFNα and IFNγ responses of CD4T_KLF2 in Pre, R, and NR groups. Two-tailed unpaired Wilcoxon rank-sum test was used.


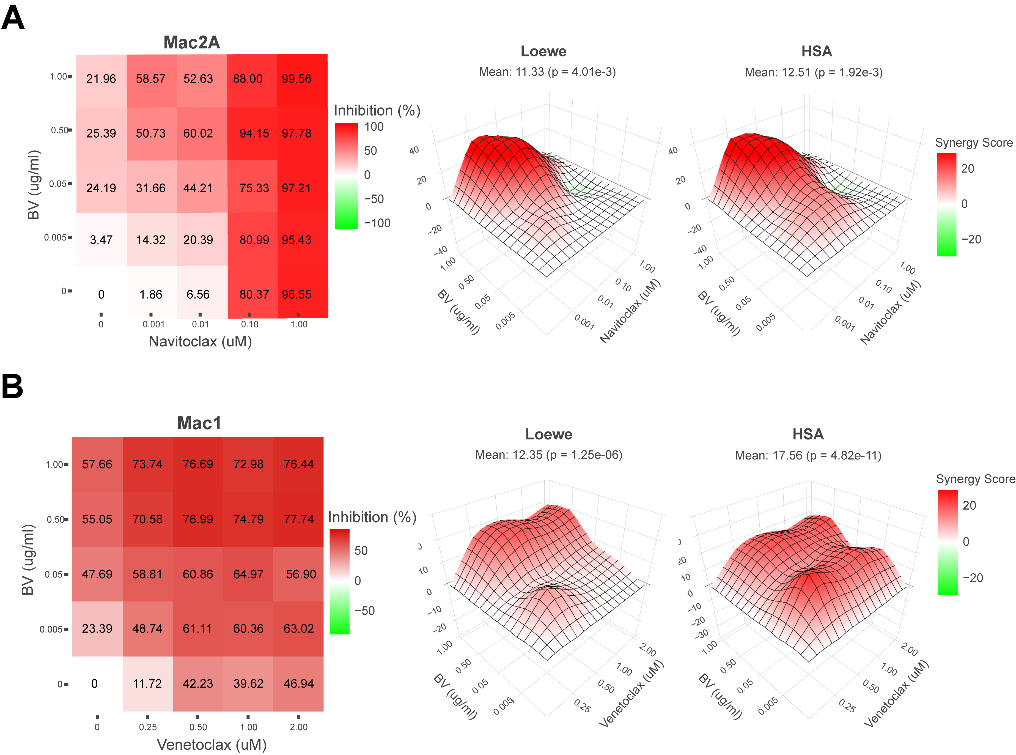


**Figure S6. BV and BCL2 inhibitors showed significant synergistic effects in CTCL.**

(A, B). Percentage inhibition of Mac2A (A) and Mac1 (B) with their synergy scores (Loewe and HSA models) shown in the middle and right panels, respectively. The synergy score represents the magnitude of synergistic (>10), addictive (-10~10) or antagonistic effects (<-10).
